# Heterodimerization of PRRSV replicase membrane proteins nsp2 and nsp3 regulates their cytoplasmic tail binding to viral RdRp domain for sgRNA synthesis

**DOI:** 10.1128/jvi.00465-26

**Published:** 2026-05-26

**Authors:** Xueyan Liu, Yunhao Hu, Qiongqiong Zhou, Peng Gao, Yongning Zhang, Lei Zhou, Xinna Ge, Xin Guo, Jun Han, Hanchun Yang

**Affiliations:** 1 State Key Laboratory of Veterinary Public Health and Safety, China Agricultural University 34752, Beijing, People’s Republic of China; 2 Key Laboratory of Animal Epidemiology of the Ministry of Agriculture and Rural Affairs, China Agricultural University 34752, Beijing, People’s Republic of China; 3 College of Veterinary Medicine, China Agricultural University 630101 , Beijing, People’s Republic of China; Loyola University Chicago - Health Sciences Campus, Maywood, Illinois, USA

**Keywords:** sgRNA synthesis, replicase membrane proteins nsp2 and nsp3, nsp9 RdRp, replication and transcription complex, PRRSV

## Abstract

Recruitment of viral RNA polymerase to replicase membrane proteins is a critical step for assembly of the replication and transcription complex (RTC) during the replication of positive-stranded RNA viruses. In this study, we report that the efficient recruitment of porcine reproductive and respiratory syndrome virus (PRRSV) RNA polymerase RdRp domain requires conformational rearrangement of replicase membrane proteins nsp2 and nsp3 via formation of heterodimeric complex (nsp2/3). Specifically, nsp2 or nsp3 interacts poorly with the nsp9 RdRp core domain (aa.500–685) in pairwise transfection, but the interaction became efficient when the two membrane proteins were co-expressed. Further analysis mapped the nsp9 RdRp-binding region to the cytoplasmic tails of both nsp2 and nsp3 and revealed that reciprocal co-expression of the transmembrane domain (TM) was sufficient to activate the binding of nsp2 or nsp3 to the nsp9 RdRp domain, whereas specific amino acid mutations within the cytoplasmic tails (nsp2 P1073A/G, N1074A/G, N1081A/G, and nsp3 R210E) could disable this interaction. The significance of nsp2/3-nsp9 RdRp interactions was revealed in studies of viral mutants, which showed that PRRSV mutants carrying the above corresponding mutations were either non-viable or severely crippled in replication and exhibited a defect particularly in viral subgenomic RNA (sgRNA) synthesis. Together, our findings highlight the importance of structure change-induced engagement of PRRSV membrane proteins (nsp2 and nsp3) with viral polymerase core domain and provide insight into the orchestrated RTC assembly in PRRSV RNA synthesis.

**IMPORTANCE:**

PRRSV represents a major threat to the global pork production, but there are no effective vaccines or antiviral drugs yet available. This report concerns the viral RTC assembly. We show that PRRSV replicase membrane proteins nsp2/3 heterodimerization induces a conformational rearrangement of their cytoplasmic tails to allow efficient interaction with nsp9 RdRp core domain. Mutations within the cytoplasmic tails that block the interactions lead to a defect in viral sgRNA synthesis. These findings add insights into the mechanisms of orderly assembly of PRRSV RTC and regulation of viral sgRNA synthesis and provide potential vulnerable targets for drug interventions.

## INTRODUCTION

During replication of positive-stranded RNA viruses, a critical step in virus life cycle is the assembly of viral replication and transcription complex (RTC). This macromolecule machinery is composed of mainly virus-encoded RNA-dependent RNA polymerase (RdRp) and various number of accessory nonstructural replicase proteins (nsps) (1–3), while its function is to direct viral RNA synthesis (4, 5). During infection, the replicase proteins are initially synthesized as large polyprotein precursors and subsequently proteolytically processed into mature forms (6–8), but the detailed mechanisms of how these nsps come together to form viral RTC has remained poorly defined for most RNA viruses. On the other hand, what is becoming clear is that the viral replicase membrane proteins play a key role in modifying host cell intracellular membranes to create a favorable environment, where other nsps are recruited to this platform (9–11). During this process, a rate-limiting step is the recruitment of core enzymes (e.g., RdRp, helicase, etc.) to RTC by viral replicase membrane proteins (12–15). This report concerns the interplay of replicase membrane proteins with RdRp of porcine reproductive and respiratory syndrome virus (PRRSV), a positive-stranded RNA virus that belongs to the family *Arteriviridae* in the order *Nidovirales* (16, 17) and a leading threat to the world swine industry for the past 30 years since its first emergence in late 1980s (18–20).

Clinically, PRRSV mainly causes reproductive failure in sows, respiratory diseases in young and fattening pigs, as well as viral or bacterial secondary infections (17, 21–23). This agent currently exhibits a worldwide distribution with an annual loss of about 0.5–0.6 billion dollars in North America and more than 130 billion RMB in China (24, 25). Unfortunately, there are currently no effective vaccines or antiviral drugs available for PRRSV control, highlighting the need for a better understanding of its replication biology to reveal potential vulnerable targets for interventions.

**Fig 1:**
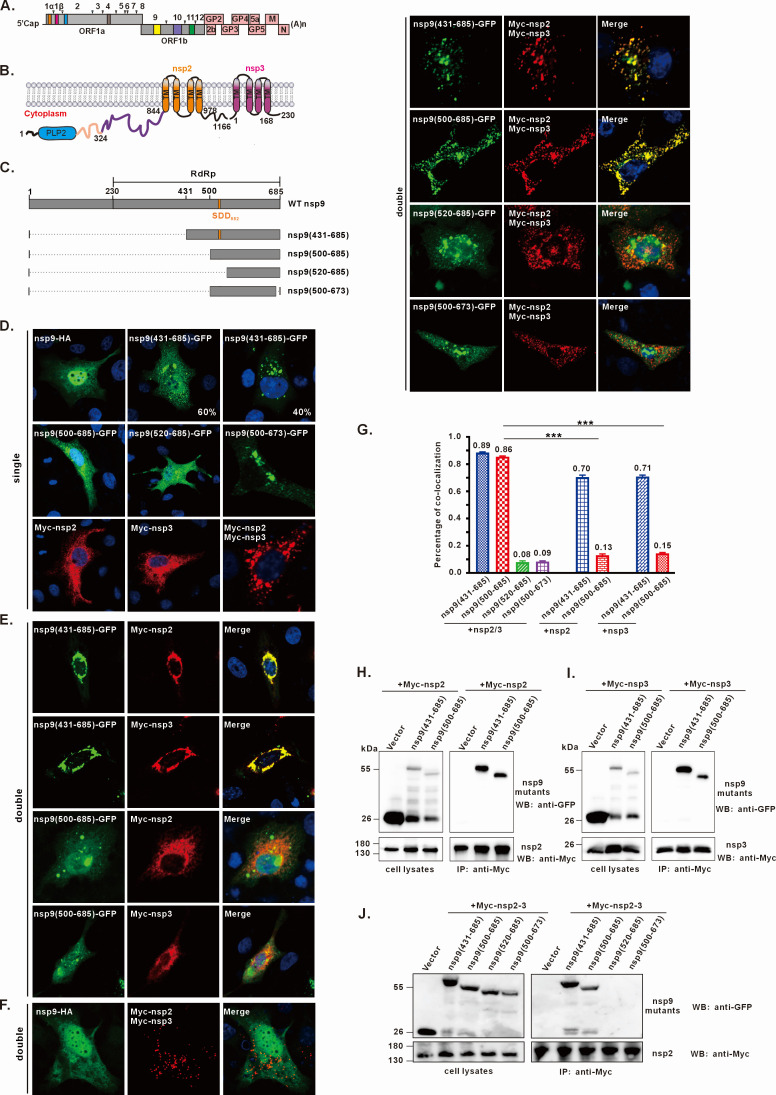
Heterodimerization of nsp2 and nsp3 promotes the binding to nsp9 RdRp domain. (**A**) Schematic diagram of PRRSV genome. Orange and red boxes: PLPα and PLPβ, papain functional domains; Blue: PL2, cysteine protease domain; Brown: 3C-like serine protease domain; Yellow: RdRp domain; Purple: helicase domain; Green: ribonuclease. (**B**) Bioinformatic topology of PRRSV nsp2 and nsp3. (**C**) Domain organization of PRRSV nsp9 and construction of its truncation derivatives. Abbreviations: RdRp, RNA-dependent RNA polymerase. SDD, the catalytic sites. (**D**) Subcellular distribution of nsp9 and its truncation mutants as well as nsp2 and nsp3 and nsp2/3 in transfected BHK-21 cells. (**E and F**) Colocalization analysis of nsp2, nsp3, and nsp9 mutants as indicated in transfected BHK-21 cells using confocal microscopy. The representative images were captured with a Nikon confocal microscope. Oil objective, 100×; zoom, 1×. (**G**) The percentages of cells showing colocalization were quantitatively analyzed from three independent experiments. Total 100 cells were usually counted for colocalization analysis. ***, *P* < 0.001. The error bars indicate standard deviations. (**H–J**) Co-IP analysis of the interactions of nsp9 mutants with nsp2 or nsp3 or nsp2/3 heterodimer. HEK293T cells were co-transfected to express the indicated protein pairs. At 24 h after transfection, cell lysates were harvested and subjected to co-IP analysis with antibodies to GFP and Myc.

The replicase proteins of PRRSV are specified by ORF1a and ORF1b (Fig. 1A). Specifically, ORF1a encodes nsp1α, nsp1β, nsp2 to nsp6, nsp2N, nsp2TF, nsp7α, nsp7β, and nsp8, of which nsp2, nsp2TF, nsp3, and nsp5 are membrane proteins, whereas ORF1b codes for viral core enzymes, including nsp9 (RNA polymerase), nsp10 (helicase), nsp11 (endoribonuclease), and nsp12, a membrane-associated protein essential for sgRNA synthesis (Fig. 1A) (6, 26–29). The past studies have revealed the existence of a complex network among PRRSV nsps (30), in which the interactions are centered between ORF1a-coded transmembrane proteins (nsp2, nsp3, and nsp5) and ORF1b-encoded enzymes (6). Notably, the interactions of the core enzymes (e.g., nsp9 and nsp10) with membrane proteins (e.g., nsp2, nsp3, nsp5, and nsp12) are subject to regulation (30–32). That is, these proteins come together during infection, but they do not interact in native condition within cells unless a portion of the protein is removed or mutated (30), suggesting an orderly assembly of PRRSV RTC.

In retrospect, the nsp9 specifies the viral polymerase that consists of an N-terminal nidovirus RdRp-associated nucleotidyltransferase (NiRAN) domain and a putative C-terminal RdRp domain (33–35), whereas the replicase proteins nsp2 and nsp3 are multiple membrane-spanning proteins (Fig. 1B) (36, 37), which, like their homologs in coronaviruses and arteriviruses, are able to form heterodimer (nsp2/3) and contribute to induction of double-membrane vesicles (DMVs) (38, 39). Quite interestingly, the interaction of nsp9 with nsp2/3 is conformation dependent as revealed by truncation mutagenesis; the full-length nsp9 does not colocalize with either nsp2 or nsp3 or nsp2/3 in co-transfected cells, but removal of the nsp9 N-terminal NiRAN domain enables the colocalization and interaction of the nsp9 C-terminal RdRp domain with these two membrane proteins (30). This report here was aimed to further explore the underlying regulatory mechanisms but with a focus on how nsp2 and nsp3 recognize nsp9 RdRp and their interaction significance. Our studies started with dissecting the minimal region of nsp9 RdRp for binding to nsp2 and nsp3 and unexpectedly reveal that a structural rearrangement via heterodimer formation is necessary for PRRSV nsp2 and nsp3 to interact with nsp9 RdRp domain and that this event is critical for viral sgRNA synthesis. The details are as follows.

## RESULTS

### Heterodimerization of nsp2 and nsp3 promotes recruitment of nsp9 RdRp core domain

The PRRSV nsp9 has a size of about 685 amino acids (aa), and its C-terminal RdRp domain (aa.431–685) has been identified as the region responsible for binding to nsp2 or nsp3 (30). Here, we went on to determine the minimum region of nsp9 RdRp domain for binding to nsp2 or nsp3 by engineering a series of truncation fragments that were expressed as GFP-fusion proteins (Fig. 1C). We tested the interactions by co-transfection assay. When expressed alone, nsp9 was mainly localized in the nucleus, while nsp9(431–685)-GFP showed characteristic discrete puncta in the cytoplasm despite nuclear presence in a portion of transfected cells (60%) (Fig. 1D). The large size of GFP tag did not appear to affect the localization of nsp9(431–685) as myc- or HA-tagged version showed similar localization pattern (Fig. S1A). This is also applicable to the subsequent multiple truncated mutants (Fig. S1B and C). In contrast, either nsp2 or nsp3 exhibited a diffusive distribution pattern in the cell cytoplasm (Fig. 1D). When co-expressed, nsp9(431–685)-GFP showed colocalization relationship with either nsp2 (70%) or nsp3 (71%) (Fig. 1E and G), displaying a distribution pattern different from that when expressed alone, but further truncation in the N-terminus, including the mutants nsp9(500–685)-GFP and nsp9(520–685)-GFP, disabled the interaction (Fig. 1E and G). Interestingly, in the Co-IP assay, nsp9(500–685)-GFP retained the ability to interact with either nsp2 or nsp3 (Fig. 1H and I), suggesting that the region nsp9 aa.500–685 is likely in an unfavorable conformation within intact cells, leading to inefficient interaction.

We next investigated whether co-expression of nsp2 and nsp3 can restore the interaction. In contrast to the diffusive cytoplasmic distribution in singly transfected cells (Fig. 1D), the co-expression of nsp2 and nsp3 leads to the induction of discrete puncta in the cytoplasm (Fig. 1D), which resembles the staining pattern in PRRSV-infected cells (30), indicating the formation of nsp2/3 heterodimer as reported previously (37). In this situation, to our surprise, the nsp2/3 complex could drastically re-localize nsp9(500–685)-GFP to form discrete puncta (Fig. 1F and G), suggesting that a structural rearrangement within nsp2 or nsp3 takes place that further exposes the binding site for RdRp. In contrast, nsp2/3 was not able to redistribute the further truncated mutants nsp9(520–685)-GFP (8%) or nsp9(500–673)-GFP (9%), in which they showed little co-localization (Fig. 1F and G). These interactions were further confirmed by co-IP assay as shown in Fig. 1J.

### The transmembrane regions mediate formation of nsp2/3 heterodimer

**Fig 2:**
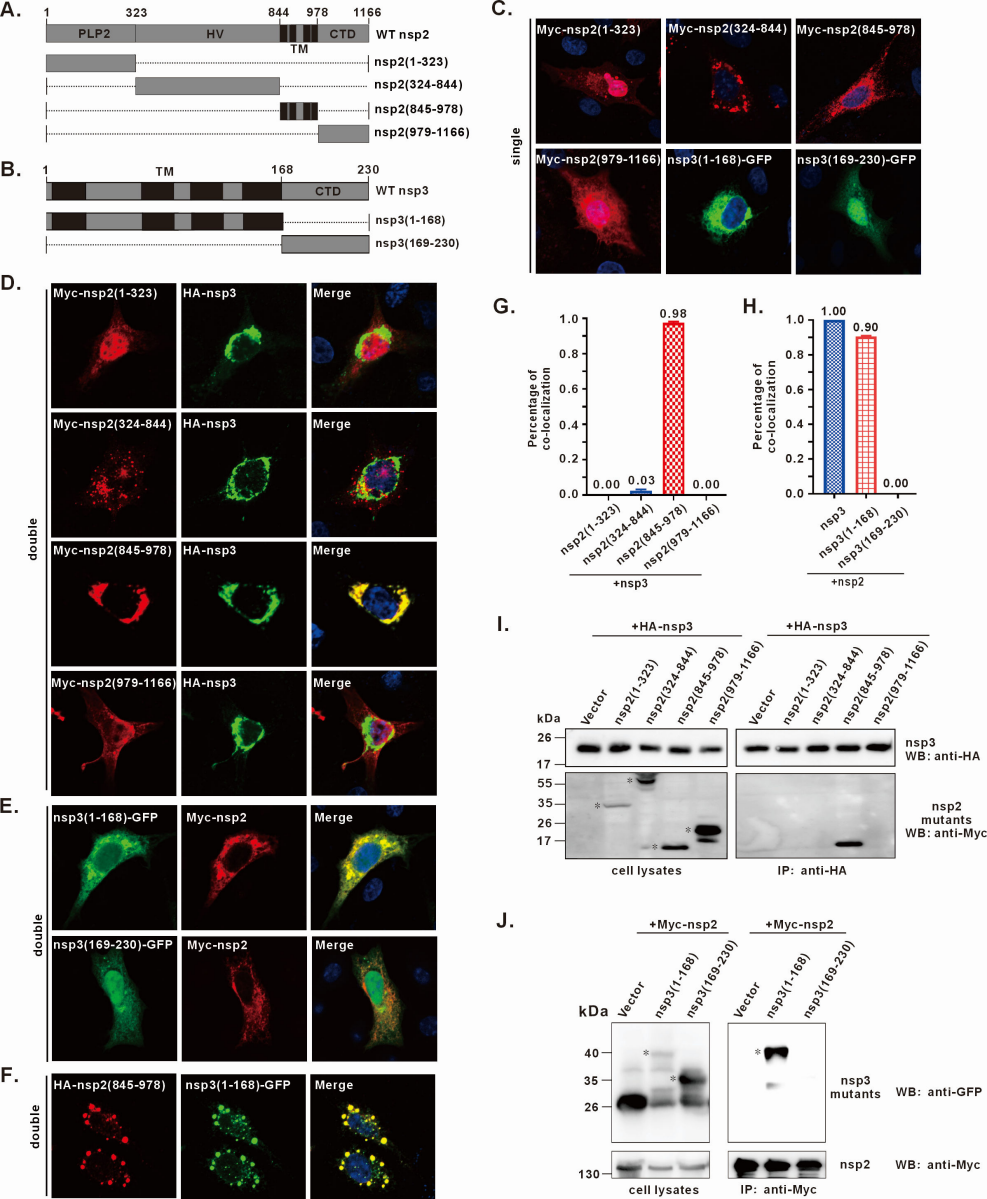
The transmembrane regions are responsible for formation of nsp2/3 heterodimer. BHK-21 cells were transfected to express the indicated protein pairs. At 18 to 24 h post transfection, the cells were fixed, stained with the appropriate antibodies to epitope tags, and examined by confocal microscopy. (**A and B**) Construction of nsp2 and nsp3 truncation mutants. (**C**) Subcellular localization of nsp2 and nsp3 mutants expressed alone. (**D–F**) Colocalization analysis of the indicated protein pairs. The representative images were captured with a Nikon confocal microscope and processed using Image J. Oil objective, 100×; zoom, 1×. (**G and H**) Quantitative analysis of colocalization percentages between the indicated proteins. Total 100 cells were usually counted for colocalization analysis. The error bars indicate standard deviations. (**I and J**) Co-IP analysis of the indicated interactions. HEK293T cells were co-transfected to express the indicated protein pairs. At 24 h after transfection, cell lysates were prepared and either analyzed by immunoblotting to measure the input expression levels or subjected to co-IP analysis interaction with antibodies to HA, c-Myc, or GFP. The asterisk indicates the specific band.

We next tried to map the key regions of nsp2/3 for heteromeric formation. Bioinformatically, PRRSV nsp2 is a multidomain-containing membrane protein (Fig. 2A), including a long N-terminal cysteine protease domain (PLP2), a hypervariable region (aa.324–844), a multiple-membrane spanning region (aa.845–978), and a C-terminal tail (aa.979–1166) according to the reference strain JXwn06 used in this study. Nsp3 has a similar topology, but with a short N-terminus (aa.1–3), a TM domain (aa.4–168), and a C-terminal tail (aa.169–230) (Fig. 2B). Accordingly, we made a series of nsp2 and nsp3 mutants, which were tagged with either Myc epitope or GFP. For the nsp2 mutants, when expressed alone, Myc-nsp2(1–323) and Myc-nsp2 (979–1166) were mainly localized into the nucleus but with diffusion in the cytoplasm, whereas the other two mutants Myc-nsp2(324–844) and Myc-nsp2(845–978) were primarily localized in the cytoplasm (Fig. 2C). In the transfected cells co-expressing with nsp3, only the fragment nsp2(845–978), corresponding to the TM region, colocalized with nsp3 (Fig. 2D and G), suggesting that the nsp2 aa.845–978 is the region for binding to nsp3. This was further confirmed by the Co-IP assay (Fig. 2I).

As for nsp3 mutagenesis, the fragment nsp3(1–168)-GFP, representing the complete TM domain, was distributed diffusively in the cytoplasm but with some nuclear membrane staining, whereas the fragment nsp3(169–230)-GFP, representing the C terminal tail, was mainly localized in the nucleus (Fig. 2C). In co-transfected cells, only the mutant nsp3(1–168)-GFP responded to nsp2, showing 90% colocalization relationship (Fig. 2E and H). When the two fragments containing the TM domain were co-expressed, nsp3(1–168)-GFP and Myc-nsp2(845–978) induced characteristic puncta formation, indicating the formation of heteromeric complex (Fig. 2F). Again, these interactions were further confirmed by Co-IP assay (Fig. 2J). Together, we conclude that the TM regions mediate nsp2/3 heterodimerization. Furthermore, since the arterivirus nsp2 and nsp3 are known to be membrane anchor proteins on DMVs that are interconnected and part of a network of modified endoplasmic reticulum (ER), we performed the co-localization analysis of nsp2 with various cellular organelle markers in the conditions of co-transfection (Fig. S2A) and PRRSV infection (Fig. S2B). We found that nsp2 does not colocalize with marker of either Golgi or Mitochondria, but shows some colocalization relationship with ER marker, in line with the idea that part of the function of nsp2/3 is to modify the intracellular membranes.

### The cytoplasmic tails of nsp2/3 mediate the binding to nsp9 RdRp domain

**Fig 3:**
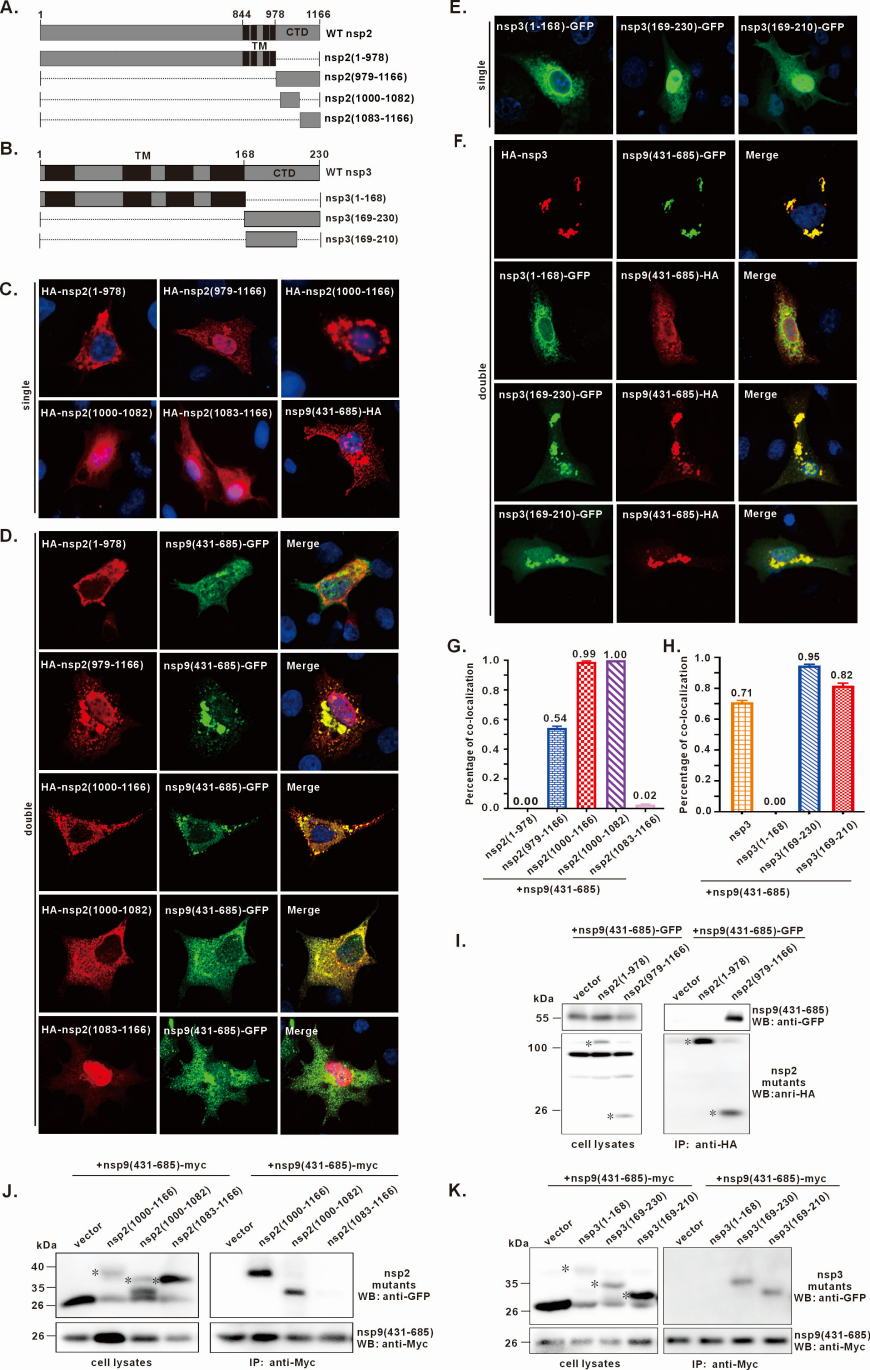
The nsp2/3 cytoplasmic tails mediate binding to nsp9 RdRp domain. (**A and B**) Construction of nsp2 and nsp3 truncation mutants. (**C**) Subcellular localization of nsp2 and nsp9 mutants. (**D**) Colocalization analysis of nsp9(431–685)-GFP with nsp2 mutants in cotransfected BHK-21 cells. (**E**) Subcellular localization of nsp3 mutants. (**F**) Colocalization analysis of HA-nsp9(431–685) with nsp3 mutants in transfected BHK-21 cells. The representative images were captured with a Nikon confocal microscope and processed using Image J. Oil objective, 100×; zoom, 1×. (**G and H**) Quantitative analysis of percentages of cells showing the colocalization between nsp9(431–685) and nsp2 or nsp3 mutants. Total 100 cells were usually counted for colocalization analysis. The error bars indicate standard deviations. (**I–K**) Co-IP analysis in transfected HEK293T cells of interaction between nsp2 (**I and J**) or nsp3 (**K**) turncations and nsp9(431–685) with antibodies to GFP, Myc, and HA. The asterisk indicates the specific band.

We utilized a series of engineered truncation mutants to map the regions of nsp2/3 for binding to nsp9 RdRp domain (Fig. 3A and B). These mutants were co-expressed with nsp9(431–685)-GFP to look for localization changes as an indication of interaction in co-transfection assay. In the initial test of two nsp2 mutants (aa.1–978, aa.979–1166), only the mutant nsp2(979–1166), which was distributed mainly in nucleus when expressed alone (Fig. 3C), responded positively with a clear redistribution, and became colocalized to nsp9(431–685)-GFP within cytoplasm. Further truncation of the N-terminal amino acids of this mutant, namely nsp2(1000–1166), enhanced the colocalization rate from about 50% to nearly 100% (Fig. 3D and G), suggesting the amino acids near TM region negatively regulate the interaction. Further delineation revealed that the region nsp2 aa.1000–1082 near the TM domain was responsible for the interaction, whereas nsp2(1083–1166) did not colocalize with nsp9(431–681)-GFP (Fig. 3D and G).

As for the nsp3 variants, when expressed alone, nsp3(1–168)-GFP was localized exclusively to the cytoplasm, whereas nsp3(169–230)-GFP and nsp3(169–210)-GFP were primarily distributed in the nucleus, with limited cytoplasmic presence (Fig. 3E). When co-expressed, nsp9(431–685)-HA did not respond to nsp3(1–168)-GFP, but rather induced a substantial redistribution of nsp3(169–230)-GFP and nsp3(169–210)-GFP from nucleus to cytoplasm, suggesting that the nsp3 C-terminal tail (aa.169–210) is involved in binding to nsp9 RdRp domain (Fig. 3F and H). This was also confirmed by co-IP assay (Fig. 3I through K). Together, the nsp2/3 complex contains two binding sites for nsp9 in their C-terminal tails, with one in nsp2 and the other in nsp3.

### Mapping the residues of nsp2/3 critical for binding to nsp9 RdRp domain

**Fig 4:**
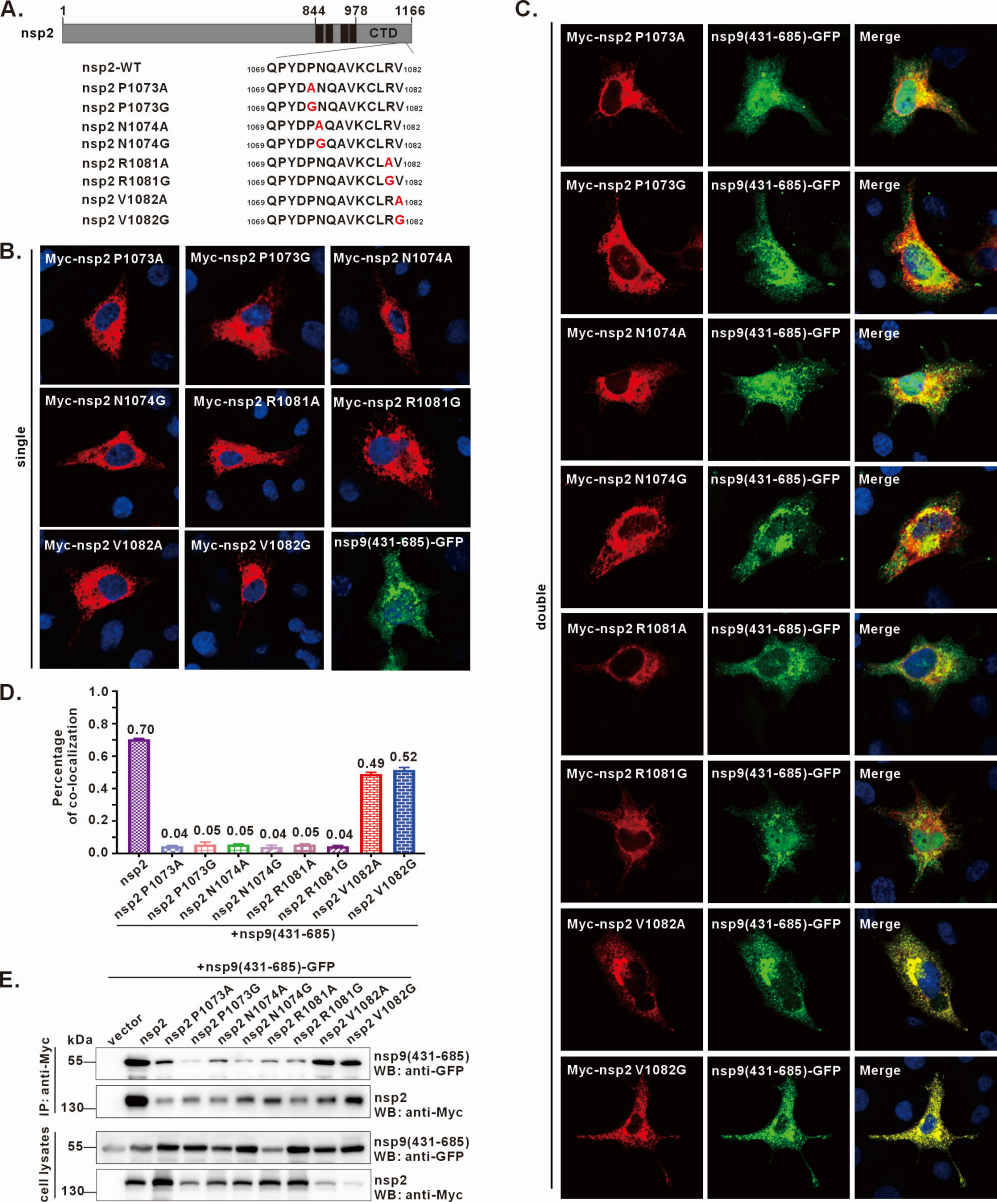
Identification of key residues within the nsp2 cytoplasmic tail for binding to nsp9 RdRp. (**A**) Sequence information of nsp2(1073–1082) and the strategy for constructing nsp2 mutants. (**B**) Subcellular localization of nsp2 mutants in singly transfected BHK-21 cells. (**C**) Colocalization analysis of nsp2 mutants with nsp9(431–685)-GFP in the co-transfected BHK-21 cells. The representative images were captured with a Nikon confocal microscope and processed using Image J. Oil objective, 100×; zoom, 1×. (**D**) Quantitative analysis of colocalization relationships in cells co-expressing nsp2 mutants and nsp9(431–685)-GFP. Total 100 cells were usually counted for colocalization analysis. The error bars indicate standard deviations. (**E**) Co-IP analysis of the interaction between nsp2 mutants and nsp9(431–685) in transfected HEK293T cells.

To identify the critical residues within nsp2 cytoplasmic tail for binding to nsp9 RdRp core domain, we performed sequence alignment of nsp2 region aa.1000–1082 across different PRRSV strains. The nsp2 region aa.1069–1082 was found highly conserved across different lineages (Fig. S3A) and targeted the deletion of the corresponding sequence led to the loss of colocalization relationship with nsp9(431–685) in co-transfected cells (Fig. S3C and D). We subsequently performed site-directed mutagenesis of this conserved region by introducing a series of double point-mutations in the context of the full-length nsp2 in a eukaryotic plasmid. We chose glycine (G) for the substitution residue in initial scanning as it represents the smallest amino acid (Fig. S3B). When co-expressed, the mutants nsp2 PN1073GG and nsp2 RV1081GG lost the ability to colocalize with nsp9(431–685)-GFP, whereas the mutations at other residues exhibited different levels of colocalization (Fig. S3D and E). In the second round scanning, single point mutations were introduced at the four positions (P1073, N1074, R1081, and V1082), and they were mutated to either A or G (Fig. 4A). When co-expressed, the mutations at the sites of P1073, N1074, and R1081 blocked the colocalization, whereas the mutations at V1082 showed relatively well colocalization with a level of about 50% (Fig. 4B through D), suggesting a less important role of V1082 in the interaction. The individual pair of interactions was further analyzed using Co-IP assay. In line with the co-localization results, both Myc-nsp2 and Myc-nsp2(V1082A/G) exhibited strong binding ability to nsp9(431–685)-GFP, whereas other mutants showed remarkably decreased interaction (Fig. 4E).

**Fig 5:**
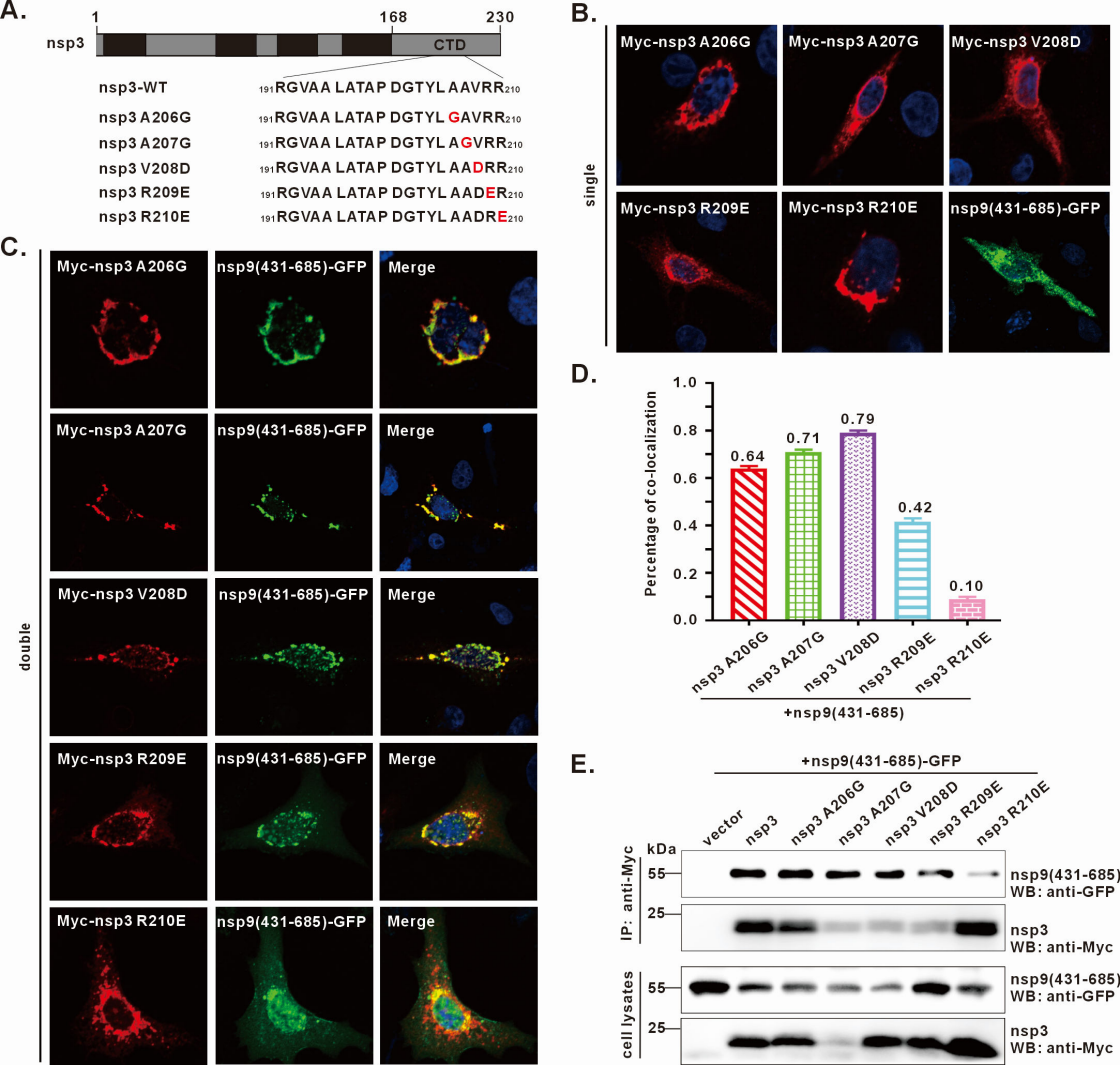
Identification of key residues within the nsp3 cytoplasmic tail for binding to nsp9 RdRp. (**A**) Sequence information for nsp3(206–210) and the strategy for constructing nsp3 mutants. (**B**) Subcellular localization of nsp3 mutants in transfected BHK-21 cells. (**C**) Colocalization analysis of nsp3 mutants with nsp9(431–685) in the co-transfected BHK-21 cells. The cells were fixed and stained with monoclonal antibodies targeting Myc. The representative images were captured with a Nikon confocal microscope and processed using Image J. Oil objective, 100×; zoom, 1×. (**D**) Quantitative analysis of colocalization relationships in cells co-expressing nsp3 mutants and nsp9(431–685)-GFP. Total 100 cells were usually counted for colocalization analysis. The error bars indicate standard deviations. (**E**) Co-IP analysis of the interaction between the nsp3 mutants and nsp9(431–685) in HEK293T cells.

Based on the lack of colocalization between nsp3(1–190)-GFP and nsp9(431–685)-HA within cells (Fig. S4A), as well as the highly conservation of the nsp3 aa.190–210 across different PRRSV strains (Fig. S4B), we performed site-directed mutagenesis targeting this specific region. For nsp3, we used a different approach as the conserved region of nsp3 cytoplasmic tail contains more charged residues. The first round of scanning was performed at every five amino acids by mutating the specific residue to its reverse property, from positive changes to negative, from hydrophobic to hydrophilic or neutral, and vice versa (Fig. S4C). Even under this dramatic alteration, the mutants 191M, 196M, and 201M showed relatively good colocalization and interaction relationship with nsp9(431–685)-GFP in about 50% of cells, while nsp3-206M_exhibited poor colocalization (about 7%) (Fig. S4D through G), suggesting that the nsp3 residues aa.206–210 play a much more important role than those in other positions and meanwhile highlighting the specificity of mutational effect. In the second round, we looked for the importance of individual residues by point mutations to generate additional mutants (A206G, A207G, V208D, R209E, and R210E) (Fig. 5A and B). The subsequent co-transfection assay identified R210 as the most critical residue necessary for efficient interaction, and the R210E mutation nearly blocked the colocalization (Fig. 5C and D). In the co-IP assay, the R210E mutation almost diminished the interaction with nsp9(431–685) (Fig. 5E). Thus, we conclude that the nsp2 residues P1073, N1074, and R1081 as well as the nsp3 residue R210 are the most critical amino acids necessary for interaction with nsp9 RdRp core domain.

### Blocking the interaction with nsp9 RdRp requires simultaneous substitutions of both nsp2 and nsp3 in transfection condition

**Fig 6:**
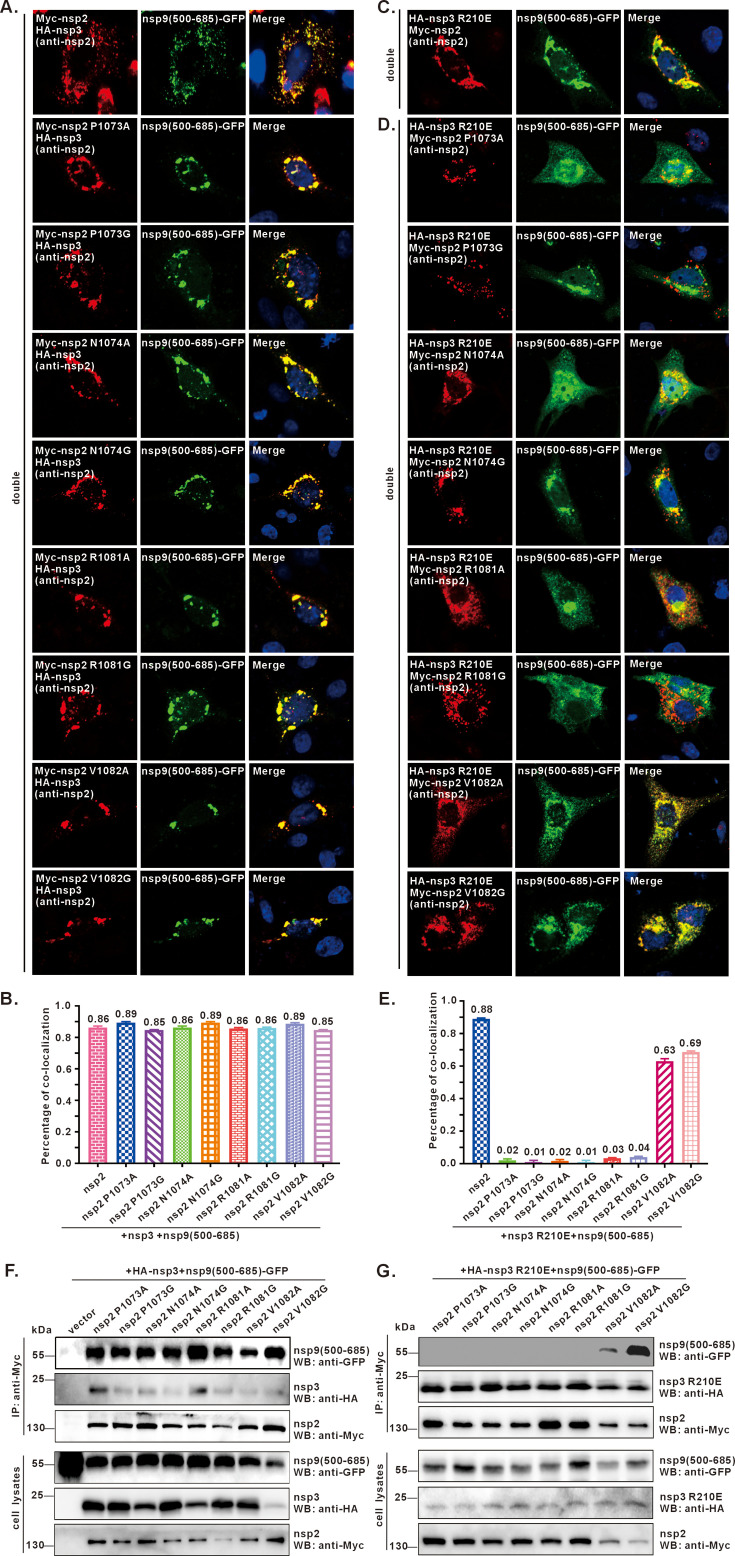
Redundancy of nsp2/3 cytoplasmic tails in binding to nsp9 RdRp in transfected cells. BHK-21 cells were transfected to express the indicated protein pairs. At 18 to 24 h post transfection, the cells were fixed, stained with the appropriate antibodies to epitope tags, and examined by confocal microscopy. (**A**) Colocalization analysis of nsp2/3 mutants and nsp9(500–685). (**B**) Quantitative analysis of colocalization relationships in cells co-expressing nsp2/3 mutants and nsp9(500–685). (**C**) Colocalization analysis of nsp2/nsp3 R210E and nsp9(500–685). (**D**) Triple expression of nsp2 and nsp3 mutants with nsp9(500–685) in BHK-21 cells. The representative images were captured with a Nikon confocal microscope and processed using Image J. Oil objective, 100×; zoom, 1×. (**E**) Quantitative analysis of colocalization relationships in cells co-expressing nsp2 mutants, nsp3 mutants, and nsp9(500–685). Total 100 cells were usually counted for colocalization analysis. The error bars indicate standard deviations. (**F**) Co-IP analysis of the interaction between the nsp2 mutants, nsp3, and nsp9(500–685) in HEK293T cells. (**G**) Co-IP analysis of the interaction between the nsp2, nsp3 mutants, and nsp9(500–685) in HEK293T cells.

The above studies showed that both nsp2 and nsp3 contain binding sites for nsp9 RdRp. We next tested the effect of point mutations on the interactions of nsp9 RdRp with heteromeric nsp2/3. We co-expressed WT nsp2 with nsp3 mutants or WT nsp3 with nsp2 mutants (Fig. S5A and B) and found that none of single mutations could block the interaction with nsp9(500–685)-GFP (Fig. 6A through C). This is expected as the heteromeric complex contains two binding sites. Thus, this promoted us to test the effect of combinatorial mutations in both nsp2 and nsp3 (Fig. S5C). In this situation, the nsp2/3 heteromeric complex carrying the mutations in both proteins failed to colocalize with nsp9(500–685)-GFP, except the nsp2 mutants V1082G and V1082A (Fig. 6D and E). Consistent results were found in the Co-IP assay (Fig. 6F and G) and in the interaction with nsp9(431–685)-GFP in the cotransfection assay (Fig. S6). Thus, the cytoplasmic tails of both nsp2 and nsp3 exhibit a seemingly redundant function in the recruitment of nsp9 RdRp domain and that mutations at both cytoplasmic tails are necessary for blocking the nsp2/3-RdRp interaction under the transfection condition.

### Co-expression with the TM domain is sufficient to activate the interaction of nsp2 or nsp3 with nsp9 aa.500–685

**Fig 7:**
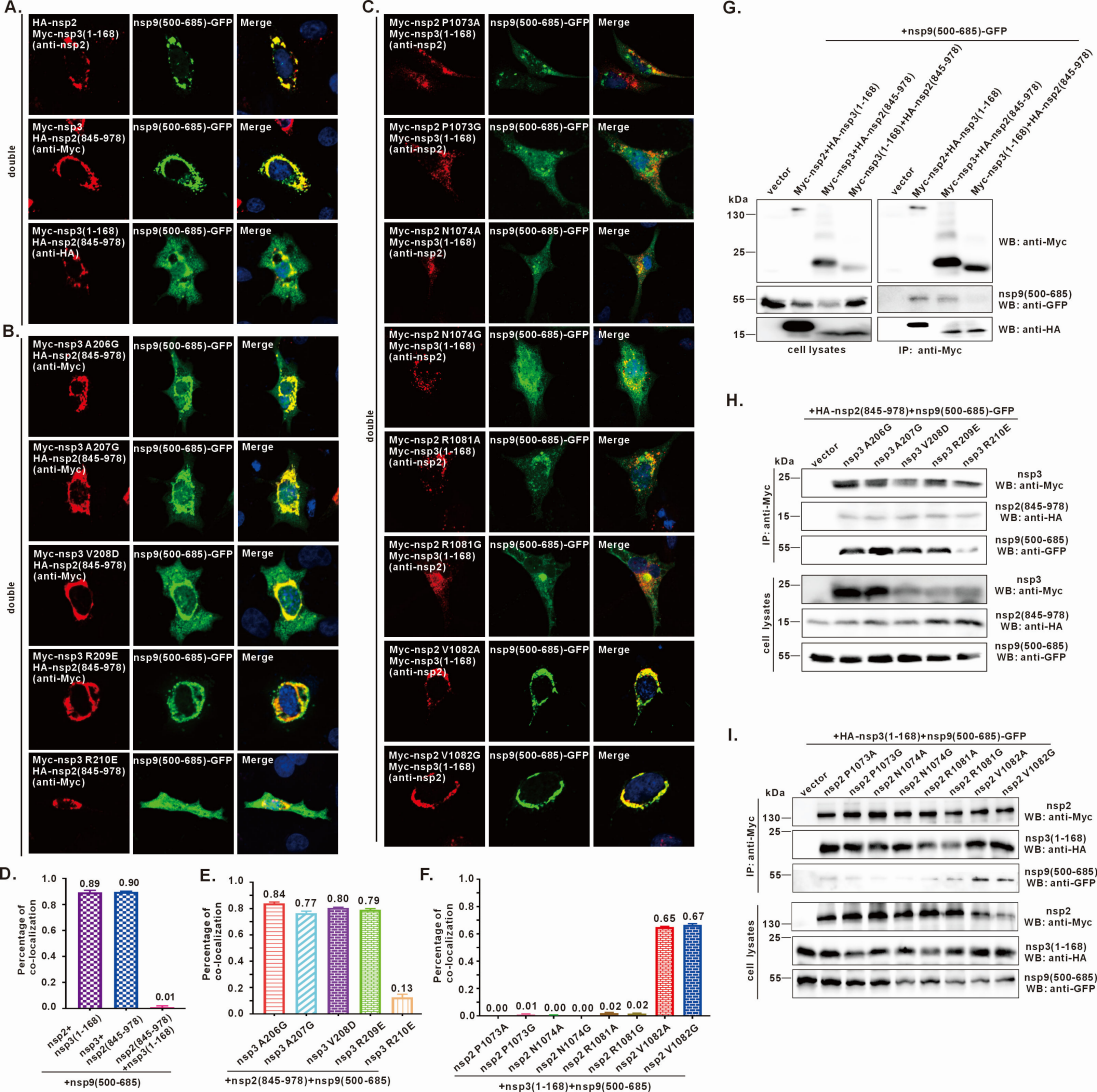
Co-expression of TM domain is sufficient to turn on nsp2/3-RdRp interaction. BHK-21 cells were transfected to express the indicated protein pairs. At 18 to 24 h post transfection, the cells were fixed, stained with the appropriate antibodies to epitope tags, and examined by confocal microscopy. (**A**) Co-localization between nsp3/nsp2 TM and and nsp9(500–685). (**B**) Colocalization between nsp2(845–978)/nsp3 mutant and nsp9(500–685). (**C**) Co-localization between nsp3(1–168)/nsp2 mutants with nsp9(500–685)-GFP. The cells were fixed and stained with monoclonal antibody targeting PRRSV nsp2. The representative images were captured with a Nikon confocal microscope and processed using Image J. Oil objective, 100×; zoom, 1×. (**D–F**) Quantitative analysis of colocalization relationships in cells co-expressing nsp2 mutants, nsp3 mutants, and nsp9(500–685)-GFP. A total of 100 cells were usually counted for colocalization analysis. The error bars indicate standard deviations. (**G–I**) Co-IP analysis of the interaction between the nsp2/nsp3 mutants and nsp9(500–685) in transfected HEK293T cells.

The above studies showed that nsp2/3 heteromeric formation can turn on the interaction with nsp9 RdRp core domain (aa.500–685). We next tested the role of TM domain in this process by co-expressing the nsp2 TM with nsp3 or nsp3 TM with nsp2. In this situation, this is only one binding site for nsp9 within the nsp2/3 heterodimer. We found that co-expression with nsp2 TM was sufficient to enable the binding of nsp3 with nsp9(500–685) as reflected by localization change and distribution patterns (Fig. 7A). As expected, the co-expression of nsp2 TM with the nsp3 mutant R210E significantly blocked the interaction, but not with other mutants A206G, A207G, V208D, and R209E (Fig. 7B and C), highlighting the specificity of mutational effect. Similarly, the nsp3 TM domain was co-expressed with various nsp2 mutants. Again, nsp3 TM was sufficient to induce the interaction of nsp2 with nsp9(500–685), and this effect could be blocked by specific mutations within nsp2 CT tail (e.g., P1073A/G, N1074A/G, and R1081A/G), but not by nsp2 V1082A/G (Fig. 7D through F). These interactions were further confirmed by Co-IP assay (Fig. 7G through I). Thus, we conclude that it is the TM domain that drives the conformation change of its corresponding binding partner (CT) to allow the binding to RdRp domain, and this takes place in a reciprocal way.

To investigate the conformation transition nature, we next tested whether the cytoplasmic tail (CT) in isolation is able to bind to nsp9 RdRp core domain. To this end, we co-expressed the nsp9(500–685)-GFP with different forms of nsp2 or nsp3 CT. As showed in Fig. S7, both nsp2(1000–1166) and nsp2(1000–1082) underwent drastic distribution and colocalized with nsp9(500–685)-GFP. As a control, nsp2(1083–1166) did not colocalize with nsp9(500–685)-GFP. In the lost-of-function assay, the above identified mutations (e.g., PN1073GG and RV1081GG) were introduced into the fragment nsp2(1,000–1,166), and it was found that these mutations could block the co-localization (Fig. S7A and B). Similar results were found for the nsp3 cytoplasmic tail (Fig. S7C and D). All together, these results indicate that the nsp2 or nsp3 CT itself is competent for binding, but they are in an unfavorable state within the full-length nsp2 or nsp3, preventing from efficient binding to RdRp core domain, whereas the nsp3/3 heterodimerization drives the conformation transition to a binding-competent state.

### The cytoplasmic tails of nsp2/3 are critical for PRRSV sgRNA synthesis

**Fig 8:**
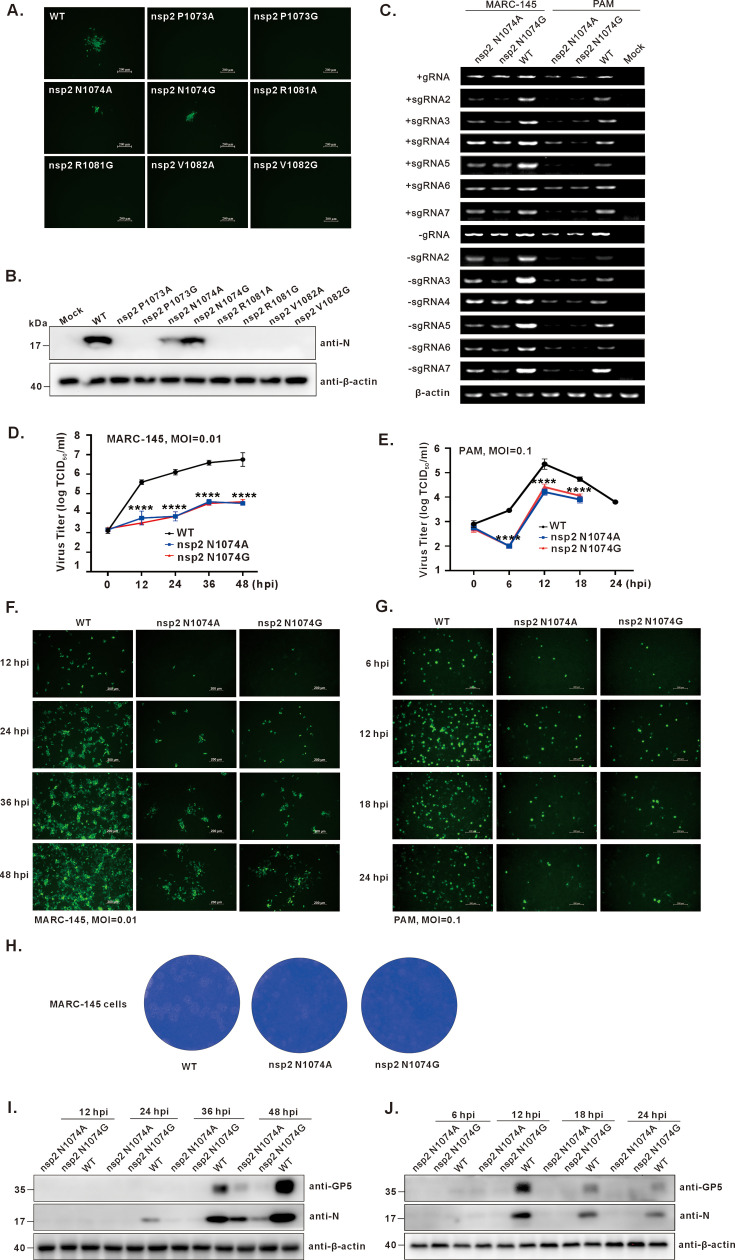
The cytoplasmic tails of nsp2/3 regulate PRRSV sgRNA synthesis. (**A and B**) Viability analysis of nsp2 mutant viruses in MARC-145 cells (**B**) and western blot with antibodies to PRRSV N protein. The transfected whole cell lysates (HEK293T-CD163) were used to infect MARC-145 cells, and at 24–36 h post infection, the cells were used for IFA or WB analysis. (**C**) A strand-specific PCR method was used to detect positive-strand RNA (+gRNA), negative-strand genomic RNA (−gRNA), positive-strand subgenomic RNA (+sgRNA), and negative-strand subgenomic RNA (−sgRNA) in both MARC-145 cells (24 h, MOI = 0.01) and PAMs (12 h, MOI = 0.1) infected with WT PRRSV or the nsp2 mutants. (**D and E**) Growth kinetics analysis of the WT and nsp2 N1074A/G viruses in MARC-145 cells and PAMs. (**F and G**) IFA analysis of the WT and nsp2 N1074A/G viruses infection and spread in MARC-145 cells at an MOI of 0.01 (**F**) and PAMs at an MOI of 0.1 (**G**) at indicated time points post-infection with antibodies to PRRSV N protein. (**H**) Plaque assay of the WT and nsp2 N1074A/G mutants in MARC-145 cells at an MOI of 0.01. (**I and J**) Western blot analysis of protein expression in MARC-145 cells at an MOI of 0.01 (**I**) and PAMs at an MOI of 0.1 (**J**).

We went on to test the mutational effect of the critical residues within the cytoplasmic tail of nsp2 or nsp3 on PRRSV replication by introducing single point mutations into the DNA-launched infectious cDNA clone of PRRSV strain JXwn06 to generate virus mutants. As a negative control, we generated a nsp9 mutant carrying a mutation at the putative catalytic site S_550_DD (nsp9 D551A) as reported previously (40). After verification by DNA sequencing, the WT and mutant infectious cDNA plasmids were transfected into the engineered HEK293T cells (HEK293T-CD163). For each mutant, three independent clones were selected for virus recovery. Of 13 mutants, most mutations were lethal to the virus, except for the mutant nsp2 N1074A/G, as evidenced by IFA and Western blot using monoclonal antibody against PRRSV N protein (Fig. 8A and B; Fig. S8, S9A and B). Interestingly, several mutations, including nsp2 V1082A and V1082G, resulted in nonviable virus despite that they did not significantly affect the nsp2-nsp9 interaction, which will be discussed later.

The defects of these mutants were explored by confocal microscopy and real-time PCR. For all the mutants, the expression of replicase proteins nsp2 and nsp9 was detectable, and they showed co-localization relationship with typical dispersed puncta in the cytoplasm (Fig. S8A, S9C and S10A). To investigate viral RNA synthesis, specific primers (Table 1) were designed to detect the synthesis of both the positive strand and negative strand genomic RNA (gRNA) as well as the subgenomic RNAs (sgRNAs) (26). The gRNAs were detected with primers targeting the coding region of nsp9, while sgRNAs were mainly amplified with primers targeting the leader-body junction region and the body sequence of the particular sgRNA. All lethal mutants showed a defect of sgRNA synthesis, suggesting the point mutations have a particular effect on sgRNA synthesis, but interestingly both viral +gRNA and −gRNA were detectable (Fig. S8B and S9D), a phenotype similar to that of the lethal mutant nsp9 D551A (Fig. S10B).

**TABLE 1:** Primers for PRRSV gRNA and sgRNA detection

Name	Sequence (5′−3′)	Purpose
gRNA-F	ACCATCACAGACTCACCATCAT	Amplification of +gRNA and −gRNA
gRNA-R	TCGCACTCACTACAAGAACCA	
sgRNA-2F	CCCTTTAACCATGAAATGGGGT	Amplification of +sgRNA2 and −sgRNA2
sgRNA-2R	GGAGCAAACCAGTCTGATGC	
sgRNA-3F	CCCTTTAACCATGGCTAATAGC	Amplification of +sgRNA3 and −sgRNA3
sgRNA-3R	TTCAAGGATCTCAGCGGCTGC	
sgRNA-4F	CCCTTTAACCATGGCTGCGTC	Amplification of + sgRNA4 and -sgRNA4
sgRNA-4R	CCATGCCTAAGGCAGCTGATG	
sgRNA-5F	CCCTTTAACCATGTTGGGGAAG	Amplification of +sgRNA5 and −sgRNA5
sgRNA-5R	GGAAACAATGTGAGTCAACAC	
sgRNA-6F	CCCTTTAACCATGGGGTCGTC	Amplification of +sgRNA6 and −sgRNA6
sgRNA-6R	GAAGGTAAAAGCACAATTCAG	
sgRNA-7F	CCCTTTAACCATGCCAAATAAC	Amplification of +sgRNA7 and −sgRNA7
sgRNA-7R	GGTAAAGTGATGCCTGACGTC	
+gRNA-RT	ATTCAGGCCTAAAGTTGGTTCA	-cDNA synthesis
-gRNA-RT	TGAGGAGCATCTACCGTCGT	+cDNA synthesis

We further characterized the replication properties of the viable mutants. The nsp2 mutants N1074A and N1074G showed significantly reduced level of viral gRNA and sgRNA in infected MARC-145 cells, an *in vitro* supporting cell line (Fig. 8C). Consequently, they were severely crippled in virus replication by 2–3 logs as measured by multi-step growth curve in both MARC-145 cells and primary porcine alveolar macrophages (PAMs) (Fig. 8D and E), the *in vivo* target cells. The virus infection and spread were also verified by IFA (Fig. 8F and G), plaque assay (Fig. 8H), and Western blot (Fig. 8I and J). The mutants overall showed much slower spread in both cell types and also exhibited significant reduction of accumulation of the major envelope protein GP5, which served as an indicator of infection. Together, the above mutational analyses highlight the critically regulatory role of the nsp2/3 cytoplasmic tails for the PRRSV sgRNA synthesis.

## DISCUSSION

Recruitment of viral polymerase to replicase membrane proteins is a critical event for RTC assembly of positive-stranded RNA viruses (7, 41, 42). Our previous studies have identified the PRRSV nsps interaction network and revealed the regulated interactions between replicase membrane proteins (nsp2, nsp3, and nsp5) and viral core enzymes (nsp9 and nsp10) (26, 30). In this report, we went on to concern the regulatory mechanisms of nsp2/3-nsp9 interactions and reveal several salient findings: (i) The nsp2 and nsp3 provide their cytoplasmic tails as the docking sites for nsp9 RdRp domain (Table S1); (ii) nsp2/3 heterodimerization induces a conformation rearrangement of cytoplasmic tails to allow efficient interaction with nsp9 RdRp core domain; (iii) nsp2/3 cytoplasmic tails critically regulate PRRSV sgRNA synthesis. An interaction model is also proposed as included in Fig. S11. The relevant significance and implications are discussed below.

Perhaps, the most unexpected finding from this study is the discovery of the significance of nsp2/3 heterodimerization. Although it is well known that PRRSV nsp2 and nsp3 are able to form a heterodimeric complex and involved in modulation of intracellular membranes (31, 38), it is not clear whether this heterodimerization will reshape their own structure and affect the interactions with their binding partners. In this report, we provide evidence for this occurrence upon heterodimer formation. The expression of either nsp2 or nsp3 alone did not result in re-distribution of nsp9(500–685), but the co-expression of both leads to its localization pattern change to characteristic cytoplasmic puncta (Fig. 1E and F), suggesting a conformational change of the cytoplasmic tail to allow efficient binding to nsp9 RdRp. Further study revealed that the TM domain itself is sufficient to induce this change (Fig. 2) as co-expression of nsp2 TM was able to activate the interaction of nsp3 with nsp9(500–685) in transfected cells (Fig. 7B and C). The same was true for the pair nsp3 TM-nsp2 (Fig. 7D and E). Meanwhile, the interaction specificity is further supported by loss-of-function assay via site-directed mutagenesis (Fig. 4 and 5). Thus, it is the heterodimerization that drives structural rearrangement of nsp2/3 cytoplasmic tails in a reciprocal manner for efficient interactions with their binding partner.

In retrospect, the interaction of nsp9 polymerase with nsp2/3 is regulated by its N-terminal NiRAN domain as removal of the region nsp9 aa.1–229 activates their interaction of the C-terminal fragment containing the RdRp domain (aa.230–685) with nsp2 or nsp3 (30). Although the mutant nsp9 aa.431–685 retained the interaction with nsp2 or nsp3, further truncation (nsp9 aa.500–685) lost this ability in transfected cells (Fig. 1). Quite surprisingly, the nsp2/3 complex formation could restore this interaction with nsp9 aa.500–685 (Fig. 1F and G). This seeming paradox is likely due to the possibility that the removal of the flanking region aa.431–499 disrupts the conformation or stability of the binding region (nsp9 aa.500–685), leading to inefficient binding in native condition, while the nsp2/3 complex via structural rearrangement somehow compensates the ability for binding. This hypothesis emphasizes a bidirectional adaptation mechanism for interaction. That is, the PRRSV nsp2/3 need to undergo conformational transition to better interact with nsp9 RdRp. In line with this notion, the cytoplasmic tails expressed in isolation, but not within the full-length form of nsp2 or nsp3, could interact or colocalize with nsp9(500–685) in transfected cells (Fig. S7). In addition, the percentage of cells showing co-localization between nsp2/3 with nsp9(431–685) is much higher (89%) than that co-expressing nsp2 or nsp3 with nsp9(431–685) (70%) (Fig. 1G). Similarly, the nsp9 polymerase is not simply recruited passively; instead, it undergoes a conformational change to achieve a structurally “compatible” state, as removal of the N-terminal NiRAN domain enables the RdRp binding to nsp2/3 (30). Although further details regarding conformation changes warrant future studies by structural biology, our studies provide strong genetic evidence for a conformation-regulated protein-protein interaction for PRRSV RTC assembly, which also has implications for understanding assembly biology of other positive-stranded RNA viruses.

The second interesting finding regards the mutational effect of nsp2/3 cytoplasmic tails on virus replication. The overall mutational phenotype on PRRSV replication is quite complex. Mutation of the critical residues (nsp2 N1073, N1074, R1081, and nsp3 R210) for nsp9 RdRp binding gave mixing results in light that the cytoplasmic tails of nsp2/3 complex play a redundant function in interaction with nsp9 in transfection condition. Of 13 mutants, only the nsp2 mutations N1074A/G gave rise to viable viruses, while other mutations were lethal (Fig. 8; Fig. S8 and S9). The lethal phenotype for the mutants nsp2 P1073A/G, R1081A/G, and nsp3 R210E suggests that the cytoplasmic tails of the nsp2/3 complex do not apparently play a redundant role in binding to nsp9 in the complicated infection condition as compared to that in transfection. In contrast to the lethal phenotype of nsp3 R210E, nsp2 P1073A/G, and R1081A/G, mutational effect of nsp2 N1074A/G, which also blocked the interaction with nsp9 RdRp core domain, is quite surprising. The underlying mechanism for this is not clear, but it may highlight the hierarchical priority of critical residues involved in interaction with nsp9 in the complex RTC network or compensatory effect by other interactions. Otherwise, what is in common for these lethal mutants is that they were all defective of viral sgRNA synthesis. Even for the viable nsp2 mutants, they also showed a significantly decreased level of sgRNA accumulation (Fig. 8C).

Surprisingly, the individual amino acid substitutions (e.g., nsp2 V1082A, nsp3 A206G, A207G, V208D, and R209E, etc.) that did not significantly disrupt the recruitment of nsp9 RdRp by nsp2 and nsp3 were also lethal to PRRSV (Fig. 8; Fig. S8 and S9). It is less likely that these mutations affect nsp2/3-induced DMV formation as the discrete puncta resembling that in PRRSV infection were induced by the transfection of PRRSV cDNA clones carrying the corresponding mutations. We postulate that these residues may be critically required for interactions with other viral proteins in the complex RTC network, a scenario that is quite different from isolated transfection condition. In any case, the mechanisms underlying the observed lethality are clearly complex, but the phenotypes of all mutants point to the conclusion that the nsp2/3 cytoplasmic tails are involved in regulating PRRSV sgRNA synthesis. This is the first time revealing a novel function for the nsp2/3 cytoplasmic tails in controlling PRRSV sgRNA synthesis. These findings also expand the regulatory network for PRRSV sgRNA synthesis to several replicase accessory proteins, including nsp1, nsp2, nsp3, nsp10, and nsp12 (26, 30).

Our findings also provide insights into the RTC assembly of PRRSV relatives. Previous studies reveal a convergent evolution between arterivirus and coronaviruses, especially on the interactions between replicase membrane proteins (nsp3, nsp4, and nsp6 for coronavirus) and the viral polymerase (nsp12 for coronavirus) (37, 39, 43). Interestingly, for SARS-CoV-2, the regions of membrane proteins nsp3 and nsp4 for recruiting the polymerase nsp12 are located within the N-terminus, rather than the C-terminus like that for PRRSV nsp2/3, whereas their CTD in involved in regulation of DMV biogenesis (44). Moreover, conformation-dependent regulation of nsp interactions also takes place. For example, SARS-CoV nsp9 are found to undergo a conformational transition from homodimeric to monomeric state to facilitate further assembly into viral RTC; mutation of nsp9 G100 and G104 residues not only affected the formation of functional nsp9 dimers (45, 46) but also impaired the interaction with nsp12 for efficient RTC assembly (47). In our studies, we provide evidence for regulated interactions between replicase membrane proteins and viral RdRp. All together, these findings highlight the intricate and dynamic properties of viral RTC assembly, which likely enables the precise regulation of viral RNA replication and transcription. In the future, a comprehensive investigation into PRRSV RTC assembly may yield precise targets for the development of antiviral therapeutics.

## MATERIALS AND METHODS

### Cells, viruses, and antibodies

BHK-21, HEK293T, HEK293T-CD163, and MARC-145 cells were cultured in Dulbecco’s modified Eagle’s medium (DMEM) (#12800-058, Gibco, NY, USA) supplemented with 10% fetal bovine serum (FBS) (#A3160801, Gibco, NY, USA) and penicillin-streptomycin at 37°C in a humidified atmosphere of 5% CO_2_. PAMs were isolated from 28-day-old SPF piglets and cultured in RPIM 1640 medium (#31800-022, Gibco, NY, USA) supplemented with 10% FBS (#A3160801, Gibco, NY, USA) and penicillin-streptomycin at 37°C in a humidified atmosphere of 5% CO_2_. Notably, HEK293T-CD163 is a modified cell line expressing the porcine molecule CD163 that exhibits an enhanced permissiveness to PRRSV, while BHK-21 cells possess the dual advantages of high transfection efficiency and permissive to PRRSV replication. The Chinese highly pathogenic PRRSV strain JXwn06 (GenBank no: EF641008) was used as the model organism in this study, and the DNA-launched infectious cDNA clone was described previously (48).

Rabbit anti-HA polyclonal antibody was purchased from MBL (561, Beijing, China). Mouse anti-Myc MAb (#60003-2-Ig), mouse anti-HA MAb (#66006-2-Ig), Rabbit anti-GFP pAb (#50430-2-AP), and β-actin MAb (#60008-1-lg) were all purchased from Proteintech (Beijing, China). Mouse anti-N (PRRSV), anti-nsp2 (PRRSV), and anti-nsp9 (PRRSV) were produced by our laboratory. Horseradish peroxidase (HRP)-conjugated goat anti-mouse pAb and HRP-conjugated goat anti-rabbit pAb were purchased from ZSGB-Bio (Beijing, China). Alexa Fluor 488-conjugated goat anti-rabbit IgG (H + L) F (ab’) 2 fragment and Alexa Fluor 568-conjugated goat anti-rabbit IgG (H + L) F (ab’) 2 fragment were purchased from Thermo Fisher. All the restriction enzymes were purchased from New England Biolabs Inc. (Beverly, MA, USA).

### Plasmid construction

The genes coding for the nonstructural proteins were successfully cloned from HP-PRRSV strain JXwn06 and subsequently engineered into the eukaryotic vectors pCMV-Myc, pCMV-HA, and pEGFP-N2, which were procured from Clontech (Mountain View, CA, USA) and stored in our laboratory. The plasmids encoding full-length nsp2, nsp3, and nsp9 have been described (30). The nsp9 truncation fragments were generated from nsp9-HA and then cloned into pEGFP-N2 to produce the corresponding mutants. For the expression of nsp2 and nsp3 derivatives, deletion and site-directed mutants were derived from full-length nsp2 or nsp3. Molecular cloning was performed through homologous recombination using the Seamless Cloning kit (#D7010M-1, Beyotime, Shanghai, China).

### Confocal microscopy

BHK-21 cells cultured on coverslips within 12-well plates were transfected with plasmid DNA using Lipofectamine 2000 DNA transfection reagent (#11668019, Invitrogen, CA, USA) in Opti-MEM medium. At 18 to 24 h post-transfection, the cells were fixed with 4% paraformaldehyde for 10 min at room temperature (RT). Subsequently, the cells were washed with 1 × phosphate-buffered saline (PBS) three times (5 min for each wash), permeabilized with 0.1% Triton X-100–2% bovine serum albumin (BSA) for 10 min, and blocked with 2% BSA for 30 min (RT). The cells were then incubated with the appropriate primary antibodies at a dilution of 1 to 1,000 for 1 h in a humid chamber at RT and then washed with PBS three times (5 min for each wash). After that, the cells were incubated with the appropriate secondary antibodies at a dilution of 1 to 1,000 for another 1 h. Nuclear DNA was stained with 4′,6-diamidino-2-phenylindole (DAPI) (62248, Thermo Fisher, MA, USA) for 10 min at RT and then washed with PBS three times (5 min for each wash). The images were captured under a Nikon A1 confocal microscope and processed using Image J.

The colocalization pattern was assessed by several measures: (i) subjective judgment of overall cellular distribution pattern of two proteins; (ii) quantitative analysis by software with respective fluorescent signals within a cell by NIS-Elements Viewer (5.21 64-bit) imaging software; (iii) statistical analysis of the proportion of cells showing overall colocalization. 100 cells were usually counted for colocalization analysis.

### Co-immunoprecipitation

HEK293T cells were cultured in 6-well plates and transfected with plasmid to express GFP-, HA-, or Myc-tagged proteins using Lipofectamine 2000 DNA transfection reagent (#11668019, Invitrogen, CA, USA) at a confluence of 70%. At 24 h post-transfection, the cells were lysed using NP-40 buffer (50 mM Tris, 150 mM NaCl, 0.5% NP-40, 0.5 mM EDTA) containing 1 mM phenylmethylsulfonyl fluoride (PMSF) and 1 mg/mL protease inhibitor cocktail (p8340, Sigma, MO, USA) for 30 min at 4°C with gentle rotation. The total cell lysates were subjected to clarification through centrifugation at 12,000 rpm for 20 min. Subsequently, cell supernatants were immunoprecipitated using anti-Myc or anti-HA MAb in conjunction with protein A/G beads (MNM-25-1000, LABLEAD, Beijing, China) overnight at 4°C with gentle rotation. The resulting immunoprecipitation pellets were collected via centrifugation, followed by three gentle washes with NP-40 buffer. The protein complexes were then boiled in 5× loading buffer for 10 min, separated by SDS-PAGE, and transferred to polyvinylidene difluoride (PVDF) membranes (ISEQ00010, Millipore, MA, USA) for Western blot analysis.

### Western blot

After being blocked with PBST (PBS with 0.05% Tween-20) containing 5% milk for 2 h, the PVDF membranes were then probed with appropriate primary antibody. Then membranes were washed three times with PBST and incubated appropriate HRP-conjugated secondary antibodies (1:10, 000) at RT. The membranes were washed and developed with enhanced chemiluminescence (ECL) detection reagents (Vigorous, Beijing, China).

### Construction of PRRSV mutant viruses

The DNA-launched infectious clone of the HP-PRRSV strain JXwn06 (pCMV-JXwn06) was under control of CMV promoter in a low-copy plasmid vector pWSK29 as described previously (49). To perform mutagenesis of nsp2, fragment A (bases 1 to 4818) containing the coding region of nsp2 was amplified from pCMV-JXwn06 to plasmid pEASY-Blunt to generate a shuttle plasmid pEASY-A. To mutate JXwn06 nsp2 nucleotides, mutagenesis PCR was performed on pEASY-A by using PfuUltra II Fusion HS DNA polymerase (#600670; Agilent Technologies). After confirmation by sequencing, mutated fragment A was digested with both restriction enzymes *SwaI*- and *BstBI* and inserted back into the infectious clone backbone to construct the final full-length deletion mutant clone. Similar strategy was used to generate nsp3 and nsp9 mutants but with both *BstBI-* and *NheI-,* or both *NheI-* and *AscI* restriction enzymes. HEK293T-CD163 cells were used for transfection and virus recovery. Briefly, the cells grown on six-well plates were transfected with plasmids of WT, nsp2 mutants, or nsp3 mutants. Cells were harvested at 2–3 days post-transfection. The cell lysates were then incubated onto fresh monolayers of MARC-145 cells to allow virus recovery. The viability of mutants was determined using immunofluorescence and Western blot analysis with antibody to PRRSV N protein.

### Detection of genomic RNA and subgenomic RNAs of PRRSV

HEK293T-CD163 cells seeded in 6-well plates upon reaching 60% confluence were transfected with 2 μg of the indicated infectious cDNA clone plasmids. At 48 h post-transfection, total RNAs were extracted with TRIzol reagent (MK05050, Magen, Guangzhou, China) according to the manufacturer’s instructions. In addition, RNA samples of rescued mutant viruses were collected from MARC-145 cells at 24 h post-infection (hpi) and from PAM at 12 hpi. Total RNA samples were heated for denaturation at 65°C for 5 min, followed by a step of digestion of input plasmid DNA at 42°C for 2 min using gDNase. Reverse transcription was performed using a FastKing RT kit (R412, Vazyme, Nanjing, China). The cDNAs of +gRNA and −gRNA were generated by RT using +gRNA-RT (primer complementing with positive strand) or −gRNA-RT (primer complementing with negative strand), respectively. Following cDNA synthesis, the template RNAs were digested by RNAse before PCR is carried out to amplify the fragment of ORF1b region using the same set of primers (gRNA-F/gRNA-R). Then, to amplify the individual sgRNAs, due to the nested feature of the sgRNAs, the downstream primers target the specific body ORF, whereas the upstream primers target the body-leader junction region of each specific ORF. The +cDNA or −cDNA were used as template, respectively, and the strand-specific PCR was subsequently performed to detect positive- and negative-strand genomic RNA (+gRNA/−gRNA) as well as positive- and negative-strand subgenomic RNA (+sgRNA/−sgRNA). The primers used for PCR analysis are listed in Table 1.

### Statistical analysis

Statistical analyses were performed using a one-way analysis of variance (ANOVA) in GraphPad Prism (version 6). All results shown are representative of three independent experiments (mean ± SEM) or of three independent experiments with similar results. Statistical significance was determined at *P* values < 0.05, indicated as *P < 0.05, ** 0.001 < *P* < 0.01, ***P < 0.001; ns indicated no statistical difference.

## Data Availability

All data generated or analyzed during this study are included in this published article and its supplemental material.

## SUPPLEMENTAL MATERIAL

Supplemental material jvi.00465-26-s0001.docx Fig. S1 to S11; Table S1.

ASM does not own the copyrights to Supplemental Material that may be linked to, or accessed through, an article. The authors have granted ASM a non-exclusive, world-wide license to publish the Supplemental Material files. Please contact the corresponding author directly for reuse.
