# Supplementary material for: Heterodimerization of PRRSV replicase membrane proteins nsp2 and nsp3 regulates their cytoplasmic tail binding to viral RdRp domain for sgRNA synthesis
Source: J Virol. 2026 May 26;100(6):e00465-26. doi: 10.1128/jvi.00465-26 (PMC13288604; doi:10.1128/jvi.00465-26)

**Supplemental data**


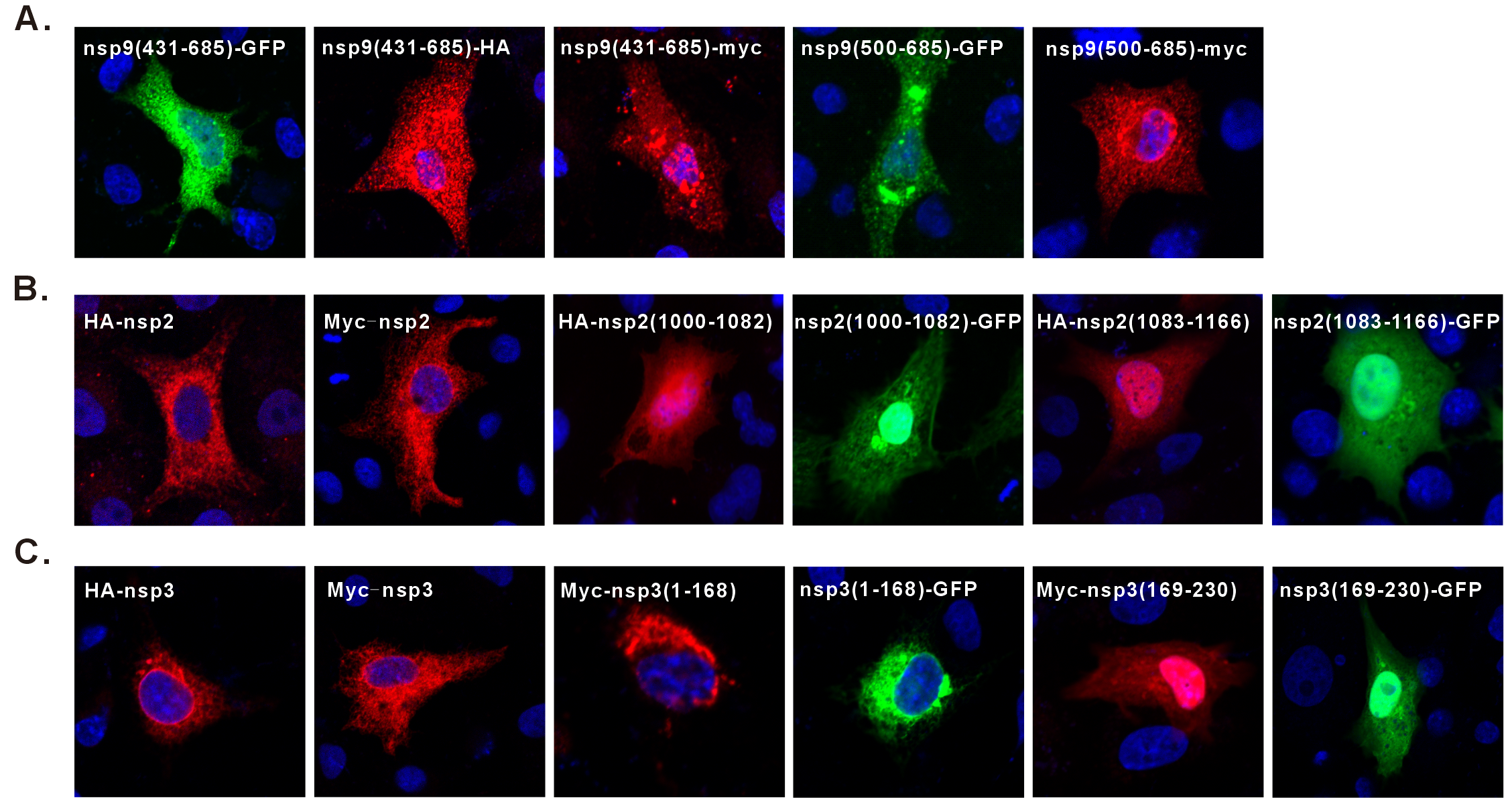


**Fig. S1 Effect of different tags on subcellular localization of nsp2, nsp3, and nsp9 and their derivatives.** Plasmids encoding nsp9 (**A**), nsp2 (**B**), and nsp3 (**C**) with different tags, as well as their derivatives, were transfected into BHK-21 cells. Protein localization within the cells was subsequently visualized using laser confocal microscopy. Oil objective, 100x; zoom, 1x.


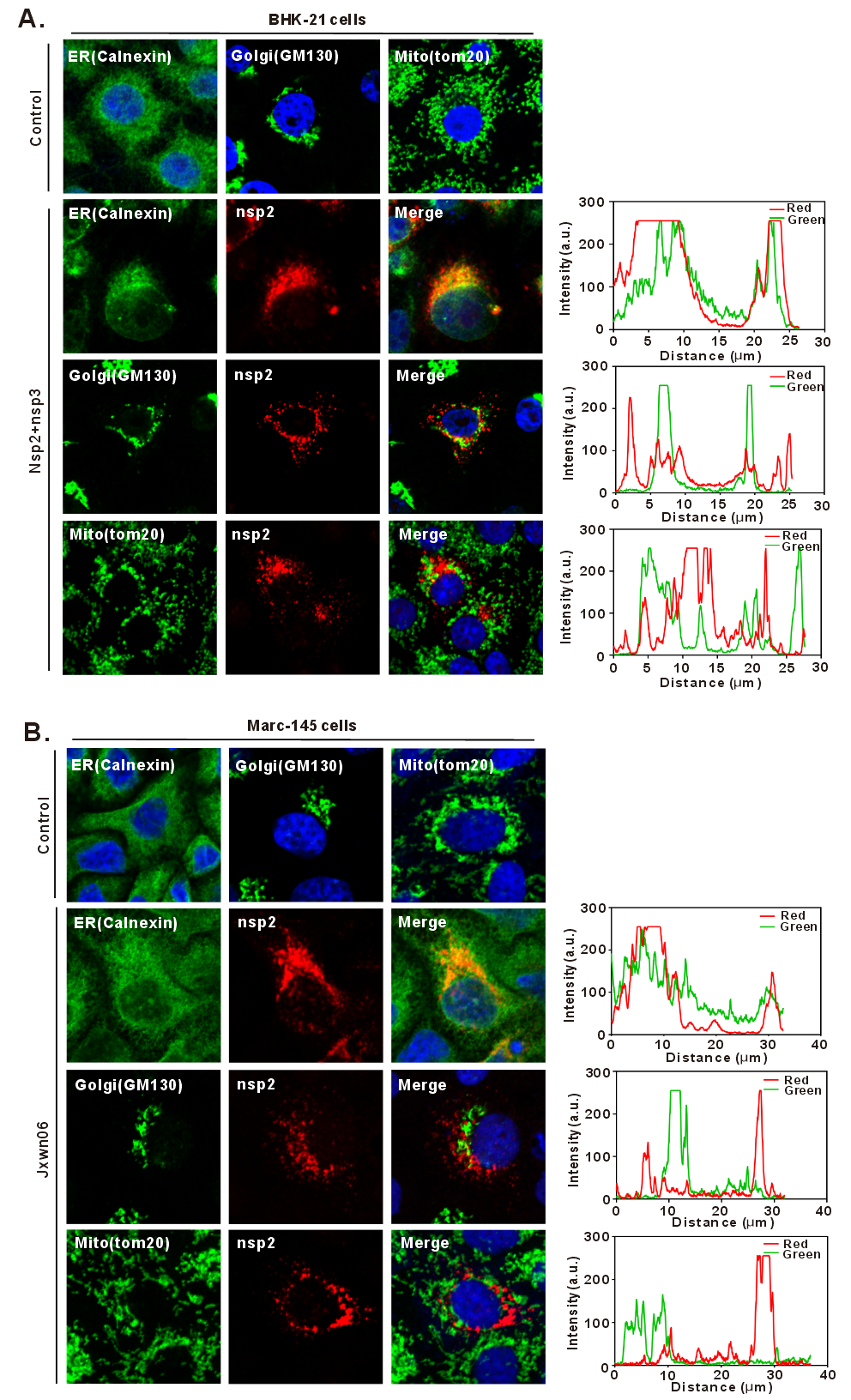


**Fig. S2 Colocalization analysis between nsp2/3 with cellular markers**. (A) BHK-21 cells were transfected to co-express nsp2/3, followed by IFA costaining with antibodies to nsp2 or Calnexin (the ER marker), GM130 (the Golgi marker), and TOM20 (the mitochondria marker) at 24 hours post transfection. (B) Similarly, the MARC-145 cells were infected with PRRSV for 24 h at an MOI of 0.1, followed by the IFA was performed. Oil objective, 100x; zoom, 1x.


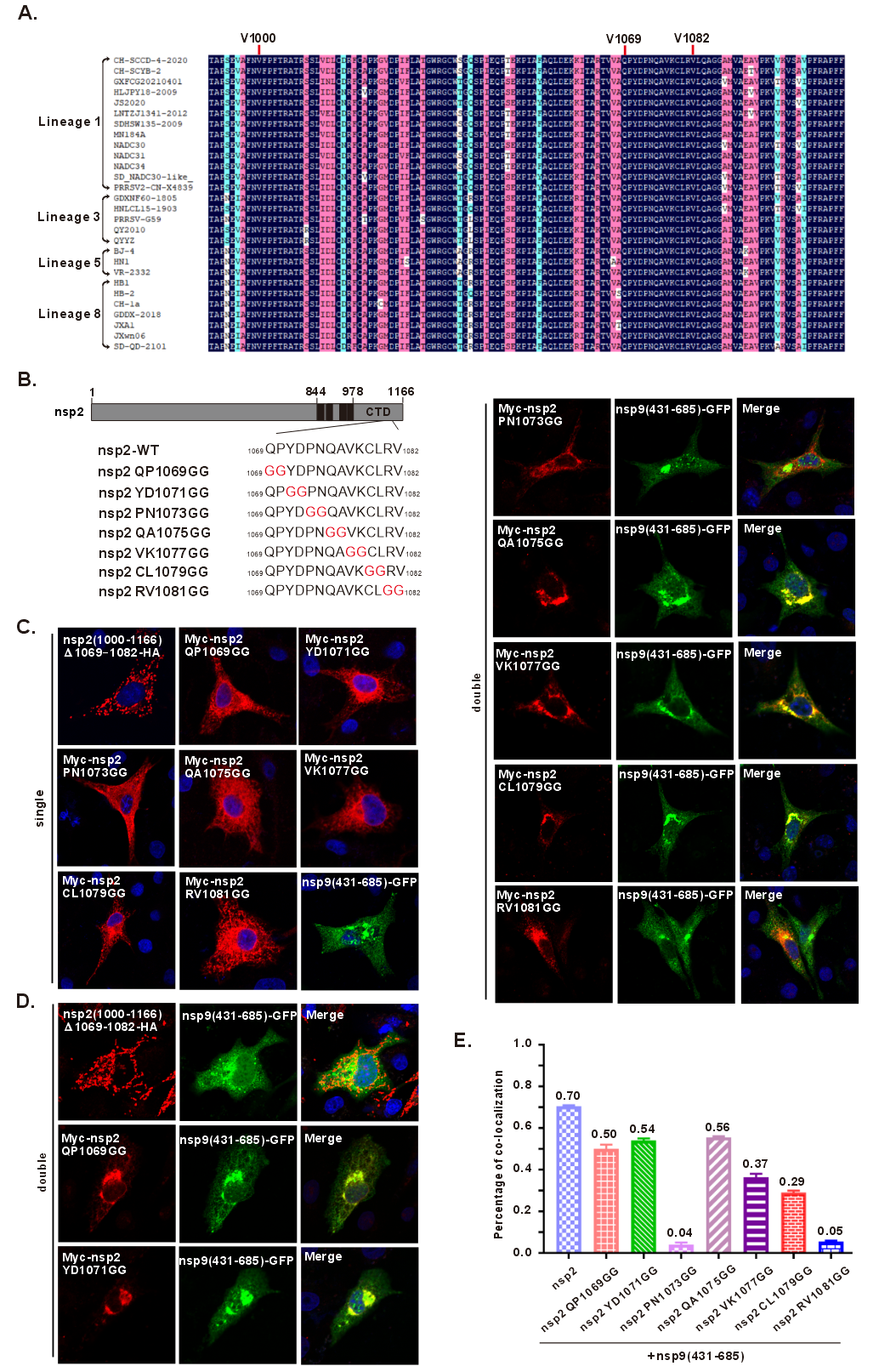


**Fig. S3 Mapping key residues of nsp2 cytoplasmic tail for nsp9 RdRp interaction.** (**A**) Alignment of nsp2 amino acid sequences between different PRRSV lineages strains. The amino acid sequences of a series of PRRSV strains were obtained from NCBI and the sequences alignment was performed using DNAMAN 6.0 software. (**B**) Construction strategy of nsp2(1069-1082) mutants. (**C**) Subcellular localization of nsp2 mutants in transfected BHK-21 cells. (**D**) Colocalization analysis of nsp2 mutants with nsp9(431-685) in the co-transfected BHK-21 cells. The cells were fixed and stained with monoclonal antibody targeting HA. The representative images were captured with a Nikon confocal microscope and processed using Image J. Oil objective, 100x; zoom, 1x. (**E**) The percentages of cells showing colocalization were quantitative analyzed in cells co-expressing nsp2 mutants and nsp9(431-685). Total 100 cells were usually counted for colocalization analysis. The error bars indicate standard deviations.


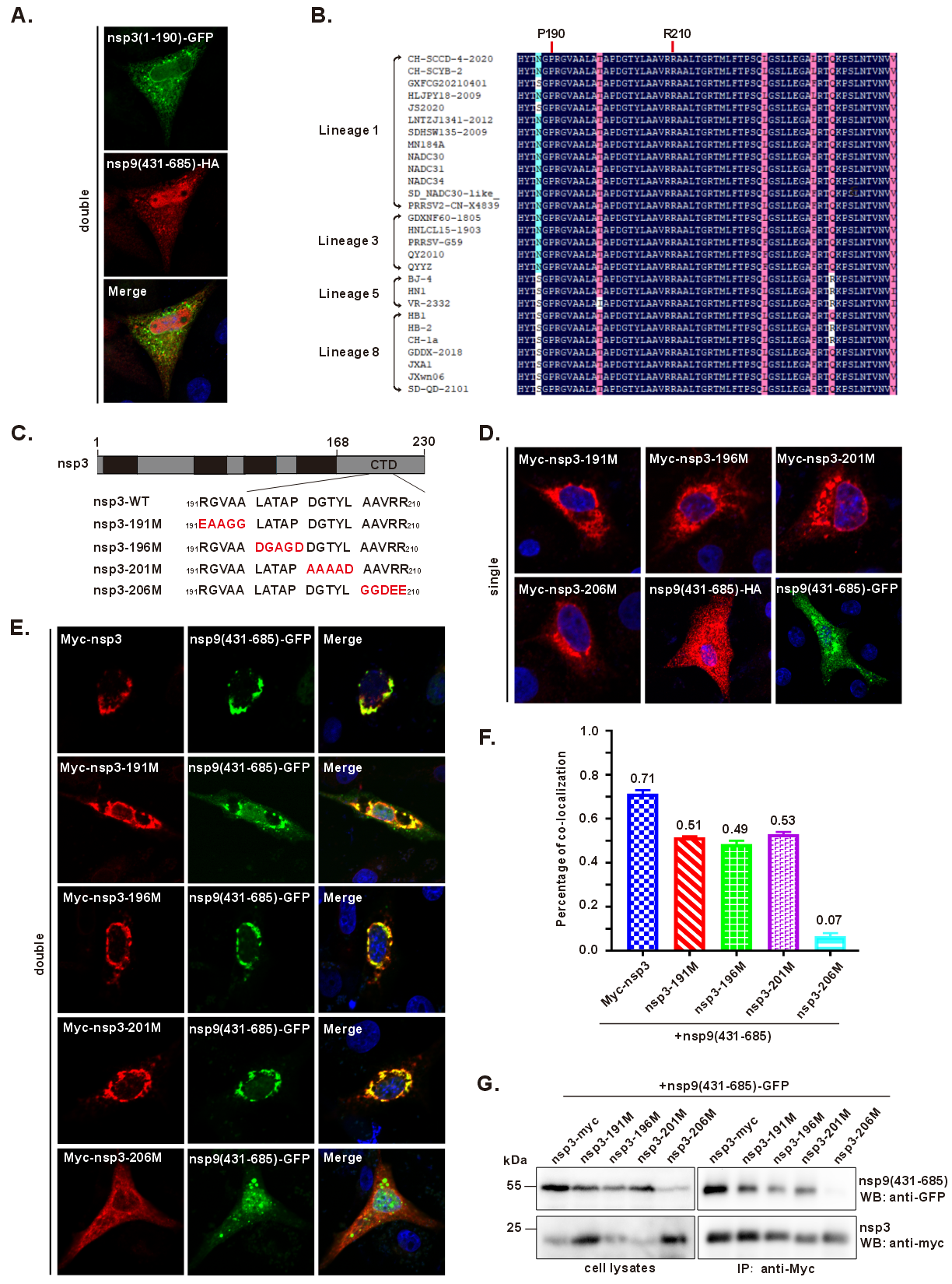


**Fig. S4 Identification of key residues within nsp3 cytoplasmic tail for binding to nsp9 RdRp.** (**A**) Colocalization analysis of HA-nsp9(431-685) with nsp3(1-190)-GFP in transfected BHK-21 cells. The representative images were captured with a Nikon confocal microscope. Oil objective, 100x; zoom, 1x. (**B**) Alignment of nsp3 amino acid sequences between different PRRSV lineages strains. The amino acid sequences of a series of PRRSV strains were obtained from NCBI and the sequences alignment was performed using DNAMAN 6.0 software. (**C**) Sequence information of nsp3(191-210) and the strategy for constructing nsp3 mutants. (**D**) Subcellular localization of nsp3 mutants in transfected BHK-21 cells. **(E)** Colocalization analysis of nsp3 mutants with nsp9(431-685) in the co-transfected BHK-21 cells. The cells were fixed and stained with monoclonal antibodies targeting Myc. The representative images were captured with a Nikon confocal microscope and processed using Image J. Oil objective, 100x; zoom, 1x. (**F**) The percentages of cells showing colocalization were quantitative analyzed in co-expressing nsp3 mutants and nsp9(431-685) cell. Total 100 cells were usually counted for colocalization analysis. The error bars indicate standard deviations. (**G**) Co-IP analysis of the interaction between nsp3 mutants and nsp9(431-685) in HEK293T cells.


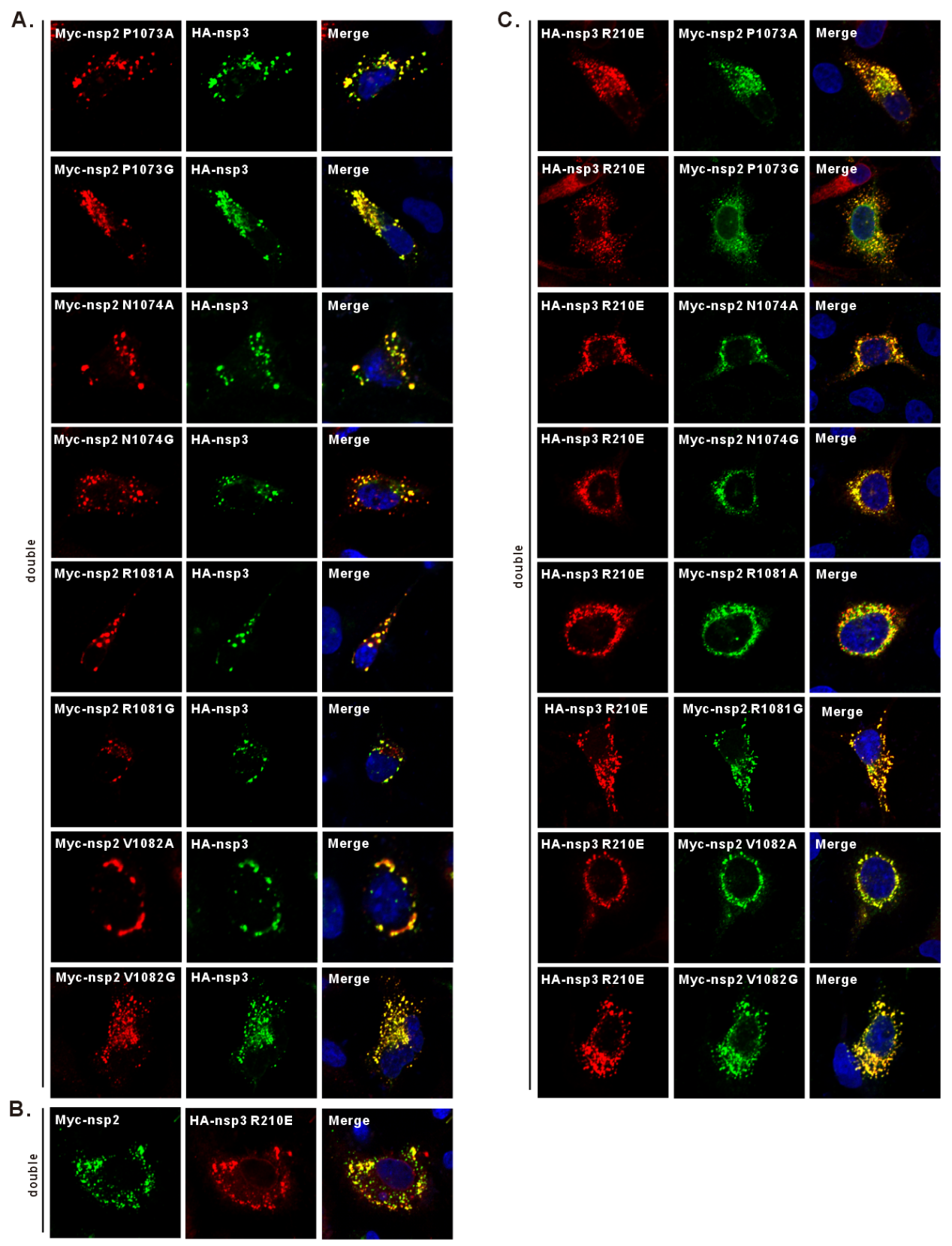


**Fig. S5 The mutations in the cytoplasmic tails do not affect nsp2/3 heterodimer formation.** (**A**) Colocalization analysis of nsp2 mutants with WT nsp3. (**B**) Colocalization analysis of nsp3 mutant with WT nsp2. (**C**) Colocalization analysis of nsp3 mutants with nsp2 mutants. The co-transfected BHK-21 cells were fixed and stained with monoclonal antibodies targeting HA or Myc epitope. The representative images were captured with a Nikon confocal microscope and processed using Image J. Oil objective, 100x; zoom, 1x.


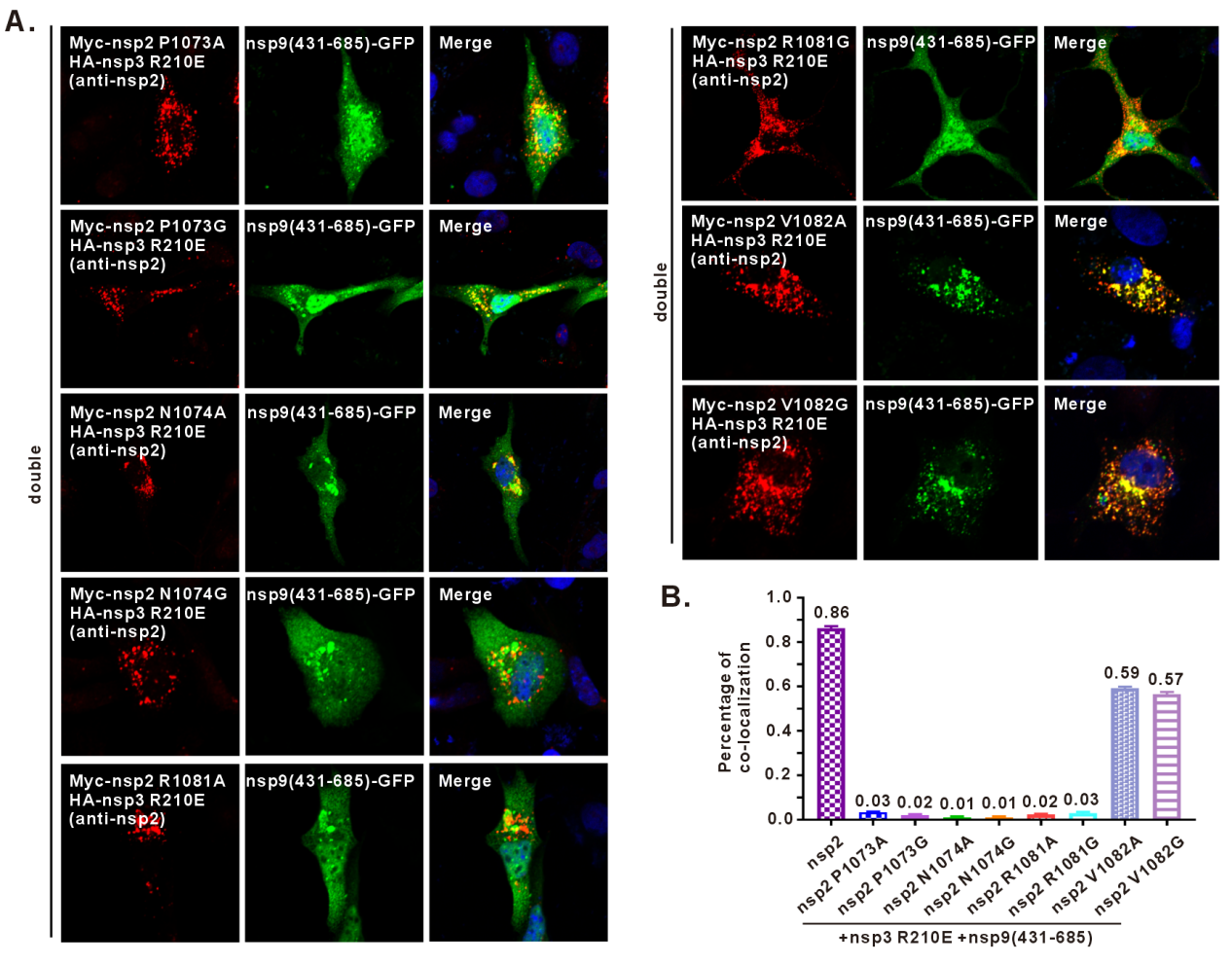


**Fig. S6 Simultaneous mutations of nsp2/3 cytoplasmic tails can block interaction with nsp9(431-685).** (**A**) Colocalization analysis between nsp2 mutants, nsp3 mutant, and nsp9(431-685). The co-transfected BHK-21 cells were fixed and stained with monoclonal antibody targeting PRRSV nsp2. The representative images were captured with a Nikon confocal microscope and processed using Image J. Oil objective, 100x; zoom, 1x. (**B**) The percentages of cells showing colocalization were quantitative analyzed in cells co-expressing nsp2 mutants, nsp3 mutant, and nsp9(431-685). Total 100 cells were usually counted for colocalization analysis. The error bars indicate standard deviations.


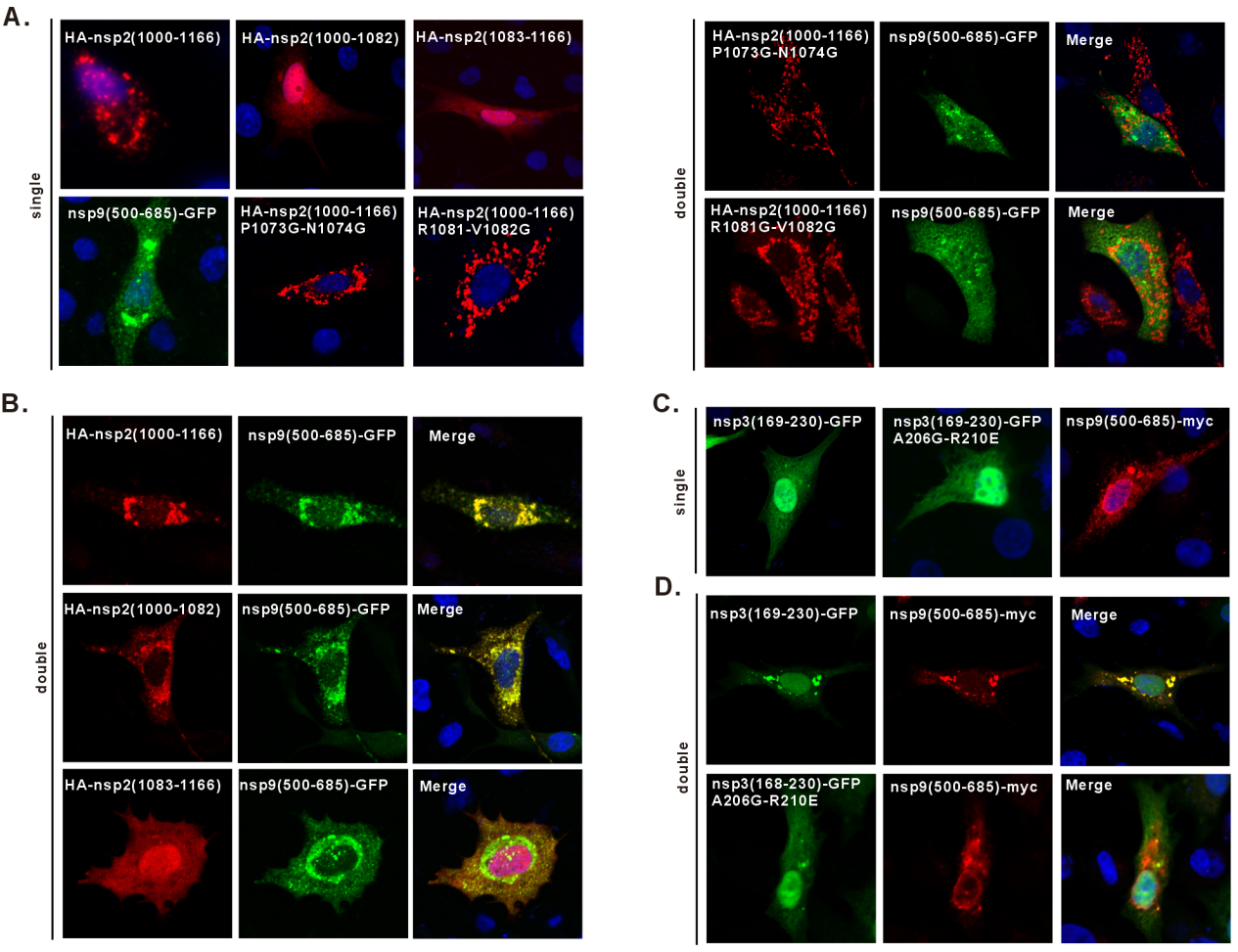


**Fig. S7 The cytoplasmic tail of nsp2 or nsp3 in isolation interacts with nsp9(500-685) in transfected cells.** (**A**) Subcellular localization of nsp2 mutants and nsp9(500-685)-GFP. (**B**) Colocalization analysis of nsp9(500-685)-GFP with nsp2 mutants in transfected BHK-21 cells. (**C**) Subcellular localization of nsp3 mutants and nsp9(500-685)-myc. (**B**) Colocalization analysis of nsp9(500-685)-myc with nsp3 mutants in transfected BHK-21 cells. The representative images were captured with a Nikon confocal microscope and processed using Image J. Oil objective, 100x; zoom, 1x.


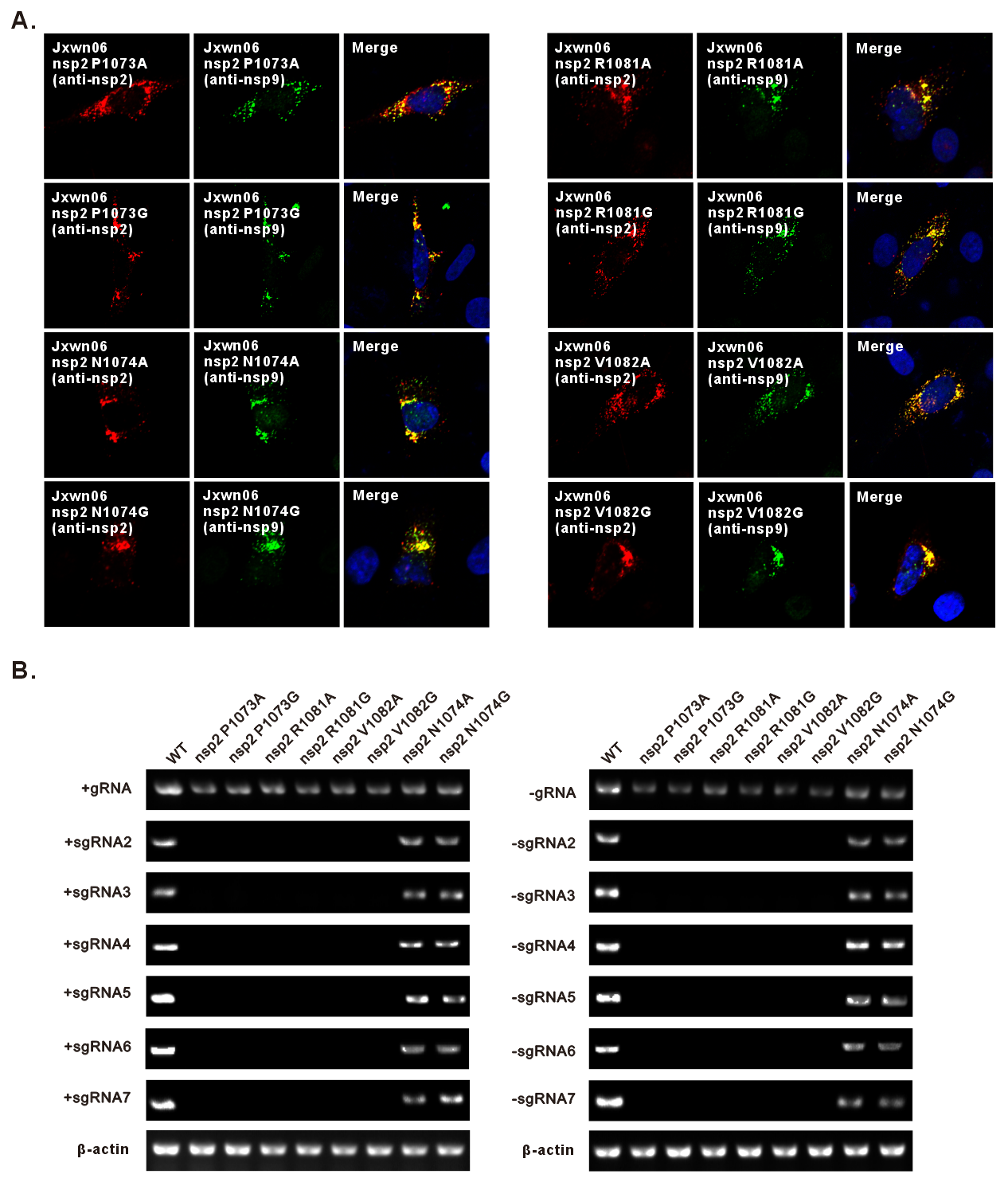


**Fig. S8 Detection of PRRSV protein and sgRNA synthesis in transfected 293T-CD163 cells.** (**A**) Colocalization analysis of viral replicase proteins nsp2 and nsp9 in HEK293T-CD163 cells transfected with the infections cDNA clone plasmid of nsp2 mutants. At 48 h post-transfection, the cells were fixed with 4% paraformaldehyde and stained with monoclonal antibodies targeting PRRSV nsp2 and nsp9 proteins. The representative images were captured with a Nikon confocal microscope and processed using Image J. Oil objective, 100x; zoom, 1x. (**B**) A strand-specific PCR method was used to detect positive-strand RNA (+gRNA), negative-strand genomic RNA (-gRNA), positive-strand subgenomic RNA (+sgRNA), and negative-strand subgenomic RNA (-sgRNA) at 48 h post-transfection from HEK293T-CD163 cells transfected with the cDNA clone plasmids for WT and nsp2 mutants.


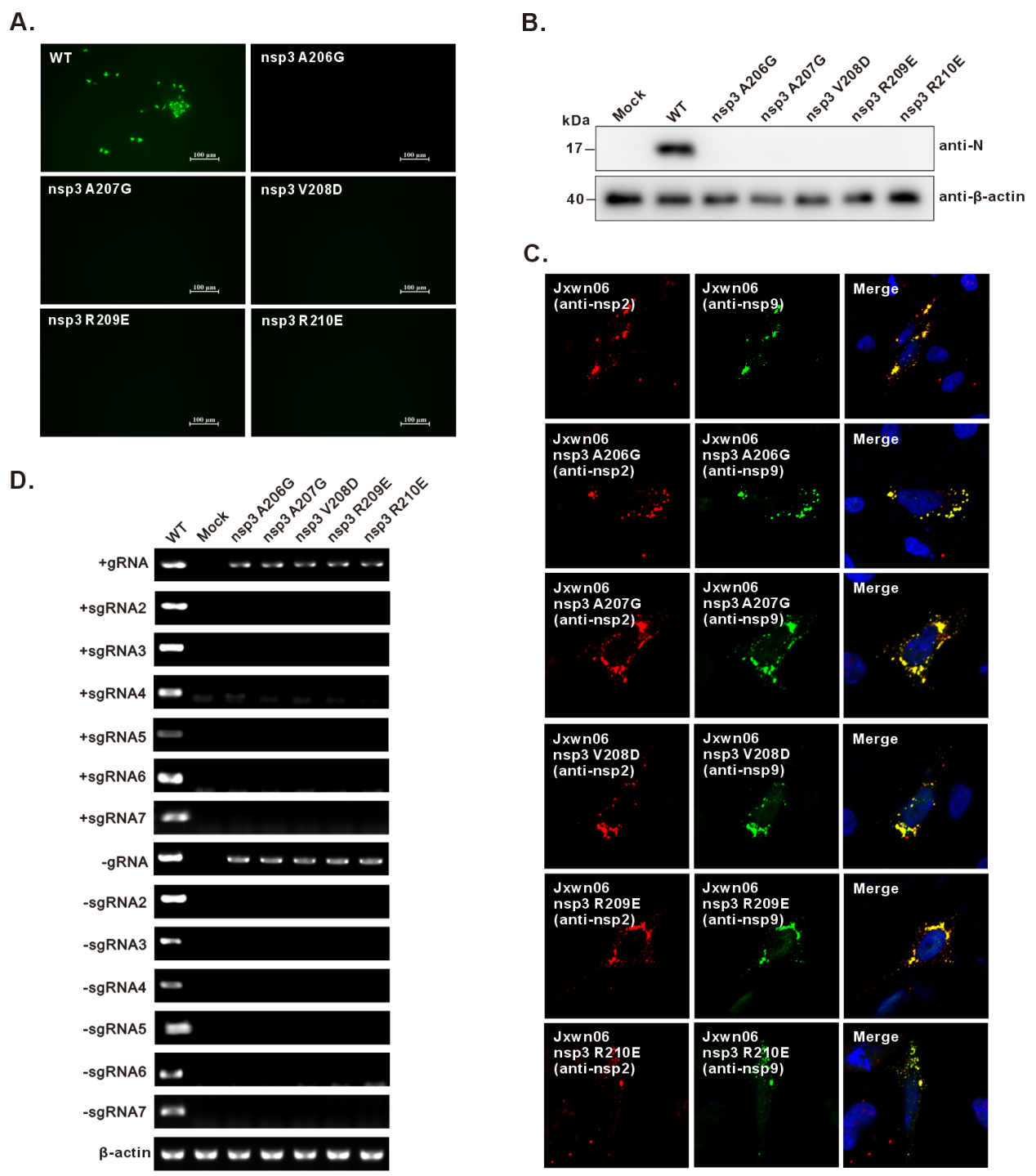


**Fig. S9 Mutational effect of specific residues of the nsp3 cytoplasmic tail on PRRSV replication.** (**A-B**) Detection of the viability of nsp3 mutant viruses in MARC-145 cells using IFA (**A**) and western blot (**B**) through targeting PRRSV N protein. (**C**) Colocalization analysis of viral replicase proteins nsp2 and nsp9 in HEK293T-CD163 cells transfected with the infections cDNA clone plasmid of nsp3 mutants. The cells were fixed with 4% paraformaldehyde and stained with monoclonal antibodies targeting PRRSV nsp2 and nsp9 proteins at 48 h post-transfection. The representative images were captured with a Nikon confocal microscope and processed using Image J. Oil objective, 100x; zoom, 1x. (**D**) A strand-specific PCR method was used to detect positive-strand RNA (+gRNA), negative-strand genomic RNA (-gRNA), positive-strand subgenomic RNA (+sgRNA), and negative-strand subgenomic RNA (-sgRNA) at 48 h post-transfection from HEK293T-CD163 cells transfected with the cDNA clone plasmids for WT and nsp3 mutants.


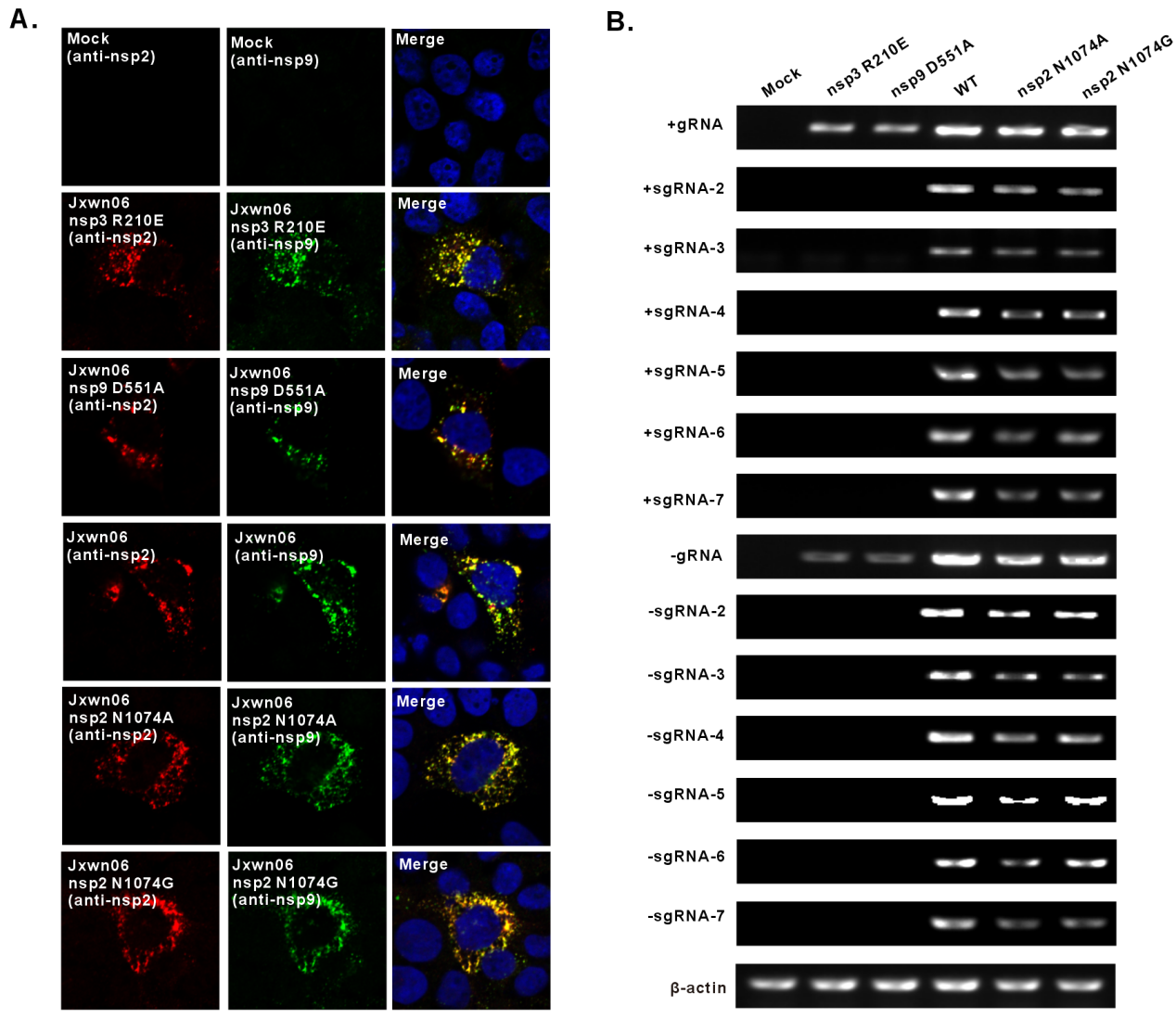


**Fig. S10 Mutation of putative catalytic sites of nsp9 (D551A) is lethal to PRRSV.** (**A**) Colocalization analysis of viral replicase proteins nsp2 and nsp9 in HEK293T-CD163 cells transfected with the infections cDNA clone plasmid of nsp9 mutants. The cells were fixed with 4% paraformaldehyde and stained with monoclonal antibodies targeting PRRSV nsp2 and nsp9 proteins at 48 h post-transfection. The representative images were captured with a Nikon confocal microscope and processed using Image J. Oil objective, 100x; zoom, 1x. (**B**) A strand-specific PCR method was used to detect +gRNA, -gRNA, +sgRNA, and -sgRNA at 48 h post-transfection from HEK293T-CD163 cells transfected with the cDNA clone plasmids for WT and nsp9 mutants. Nsp2 and nsp3 mutants were used as the control.


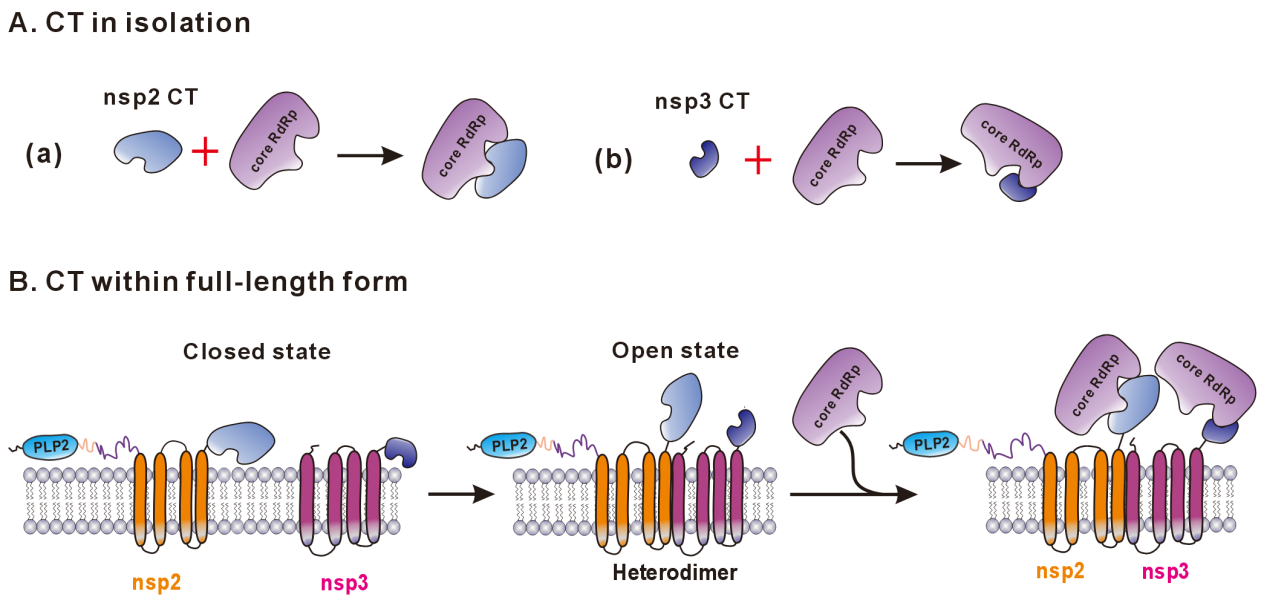


**Fig. S11** **Proposed model for nsp2/3 interaction with nsp9 RdRp core domain.** The isolated cytoplasm tails of nsp2 and nsp3 bind to the nsp9 core RdRp domain in the intact cells (**A**), but do not interact in the full-length form, unless the nsp2/3 heterodimerization takes place (**B**), which induces a conformation rearrangement of cytoplasmic tails to allow efficient interaction with nsp9 RdRp core domain.

**Table S1. Summary of all nsp2 and nsp3 mutants utilized in this study**


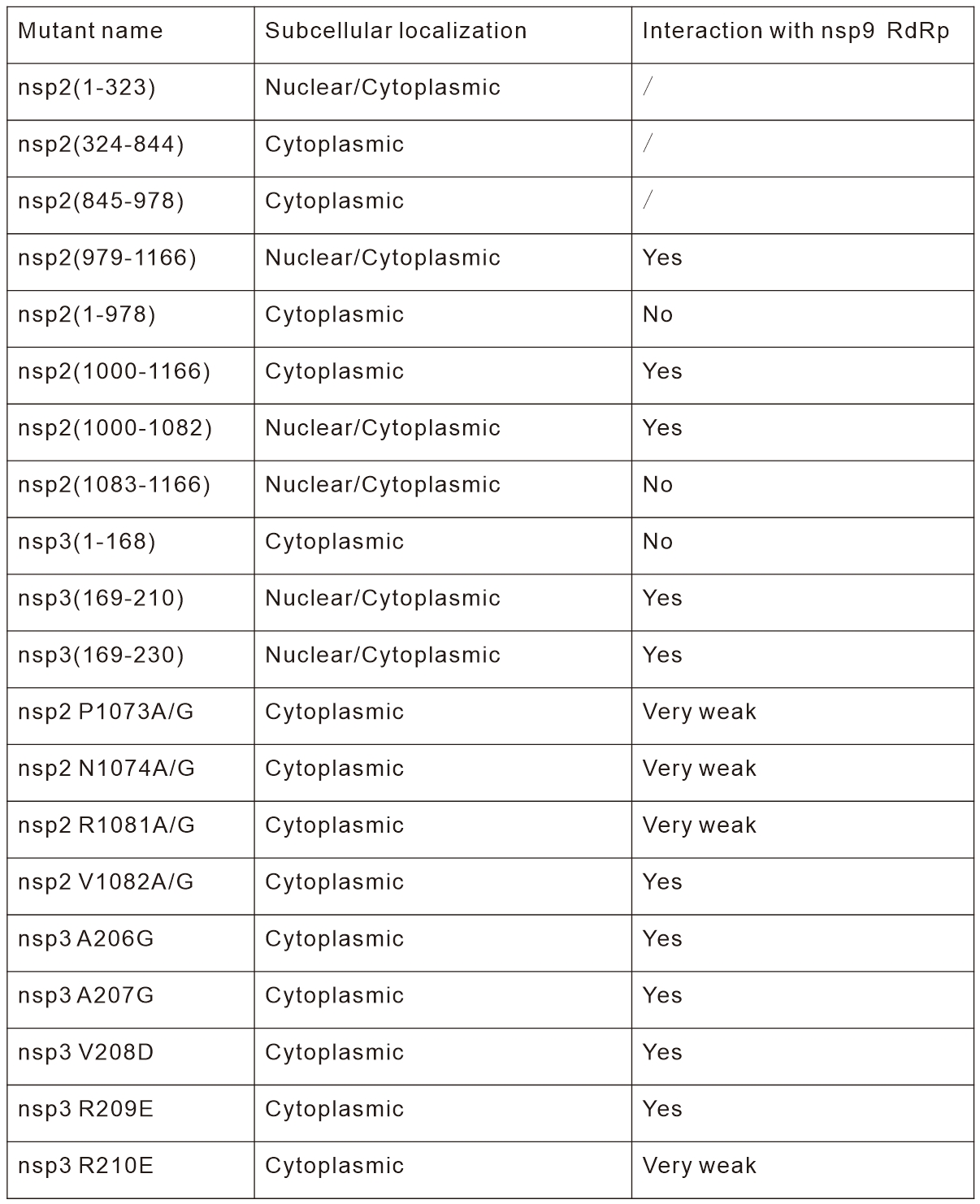

Supplement: Supplemental material — Fig. S1 to S11; Table S1. [file jvi.00465-26-s0001.docx]
